# Exploring the relationship between land use/land cover and apparent temperature in China (1996–2020): implications for urban planning

**DOI:** 10.1038/s41598-024-53858-8

**Published:** 2024-02-08

**Authors:** Han Ding, Qiuru Ren, Chengcheng Wang, Haitao Chen, Yuqiu Wang

**Affiliations:** College of Environmental Science and Engineering, Nankai University, Tianjin, 300350 China

**Keywords:** Apparent temperature, LULC change, Thermal comfort, Urban planning, Climate sciences, Environmental sciences

## Abstract

In recent decades, rising air temperatures (AT) and apparent temperatures (AP) have posed growing health risks. In the context of China's rapid urbanization and global climate change, it is crucial to understand the impact of urban land use/land cover (LULC) changes on AP. This study investigates the spatial distribution and long-term variation patterns of AT and AP, using data from 834 meteorological stations across China from 1996 to 2020. It also explores the relationship between AT, AP, and LULC in the urban core areas of 30 major cities. Study reveals that AT and AP exhibit overall high spatial similarity, albeit with greater spatial variance in AP. Notably, regions with significant disparities between the two have been identified. Furthermore, it's observed that the spatial range of high AP change rates is wider than that of AT. Moreover, the study suggests a potential bivariate quadratic function relationship between ΔT (the difference between AT and AP) and Wa_ratio and Ar_ratio, indicating the presence of a Least Suitable Curve (LSC), ${\text{W}}_{{\text{a}}} \_{\text{ratio}} = {0}{{.263(}} \pm {0}{{.0269)}} - {0}{{.437(}} \pm {0}{{.0417)}} \times {\text{Ar}}\_{\text{ratio}}$. Urban LULC planning should carefully avoid intersecting with this curve. These findings can provide valuable insights for urban LULC planning, ultimately enhancing the thermal comfort of urban residents.

## Introduction

Over the past few decades, ongoing climate change characterized by global warming has resulted in an increase in extreme temperature events, leading to heightened exposure to these exceptional temperature conditions among human populations worldwide^1,2^. Consequently, the impacts of extreme hot and cold weather on both ecological environments and human societal development have grown increasingly severe. Extreme temperature events stand as the principal contributors to human morbidity and mortality associated with climate change^3–9^. For instance, scorching summer heat can lead to heatstroke, heat exhaustion, and skin rashes^1^, while frigid winter temperatures can exacerbate stroke-related mortality. Both extreme high and low temperatures exert discernible influences on cardiovascular diseases^4^, and so forth. Research indicates that fatalities attributed to extreme cold and heat events account for 7.71% of total mortality^4^. Therefore, the assessment and causal analysis of extreme temperatures become paramount.

AT is commonly utilized by individuals as a metric for assessing local thermal conditions. Nonetheless, when the multifaceted impact of various factors, such as wind chill and humidity, is considered, human health faces a more ominous threat compared to the impact of individual temperature factors^8,10–13^. In environments with higher humidity under the same temperature, individuals often perceive a higher level of heat^14^, whereas windy conditions feel colder than still air. In 1984, Robert G. Steadman introduced the concept of apparent temperature (AP), which integrates AT and relative humidity (RH) into a unified index^15^. This index was further refined in 1994 to encompass wind speed and solar radiation^15,16^. As a human-centric composite meteorological index, AP has the capacity to simultaneously capture both thermal comfort and the natural climatic state under corresponding weather conditions^5,17^. It stands as an effective indicator for investigating the impact of meteorological conditions on human health and has found widespread application in the fields of meteorology and public health^18,19^.

Numerous factors influence urban thermal environments, such as increased carbon dioxide emissions, leading to the greenhouse effect and elevated urban temperatures^20^. Furthermore, the proliferation of artificial structures and the reduction of green spaces during the urban land expansion process ^21,22^ also modify urban thermal environments. These developments underscore the significant impact of urbanization on urban thermal environments. Over the past few decades, substantial population growth worldwide has accelerated urbanization. Urbanization intensifies energy consumption, for instance, for winter heating and vehicular emissions, which contribute to greenhouse gas emissions and heat production. Nevertheless, urban land expansion, or more specifically, changes in urban land use and land cover (LULC), remains the most direct pathway through which urban development influences regional temperatures^23–26^.

Different land use types exhibit distinct characteristics that influence their temperature patterns^27,28^. For instance, water bodies characterized by their high heat capacity and low thermal conductivity, exert a notable influence on urban temperatures. Green spaces can generate cool island effects by blocking solar radiation and reducing emissivity and thermal inertia. In contrast, artificial surfaces have strong heat-storage capacity, impede atmospheric motion, and absorb more solar radiation, resulting in reduced evaporative cooling. As a result, replacing water bodies and green spaces with impermeable surfaces such as buildings, roads, and other infrastructure, as observed in urban land expansion, has a direct impact on urban AT and AP^1,29^. The first written record of urban areas exhibiting higher temperatures than surrounding rural areas dates back to 1833^30^. Manley^31^ introduced the concept of the Urban Heat Island (UHI), which is the quintessential representative of how LULC changes affect thermal environments. In the investigation conducted by Liu et al.^32,33^, contrasting impacts of precipitation deficits on heat stress were observed in the northeastern and southern regions of China. This observation suggests potential variations in the influence of RH on the assessment of heat stress across distinct geographical areas. Additionally, it hints the possibility that LULC changes may play a role in influencing urban thermal comfort.

Driven by rapid urbanization, China has witnessed significant changes in urban LULC that have substantially impacted urban thermal environments over recent decades^11,19,34^. Studies have revealed that urbanization intensifies the frequency of extreme climate events, and its influence on AP is more profound than that on AT^1,19^. This has motivated our exploration into the influence of LULC changes on the disparities between AT and AP. Regionally, the urbanization effect is particularly pronounced in densely populated and economically developed urban areas such as the Beijing-Tianjin-Hebei region, the Yangtze River Delta, and the Pearl River Delta^11,34,35^. Overall, compared with southern cities, AP in northern cities is more sensitive to urbanization^1,25,26^. This influence has the potential to exacerbate thermal discomfort among urban residents, heightening regional health risks and simultaneously posing a threat to urban ecological environments^11,19,34^. In recent years, research on the influence of LULC changes on urban thermal environments has increased, albeit primarily focusing on the single metric of AT^27,36,37^, with limited exploration of comprehensive indices like AP, and an even scarcer examination of the impact of LULC changes on the disparities between AT and AP.

In light of these concerns, this study employs daily meteorological data from 834 stations across China to analyze the spatiotemporal distribution and trends of AT and AP from 1996 to 2020. Simultaneously, we further examine multi-year land use and land cover (LULC) data for the core areas of 30 developed cities, quantifying their influence on contemporaneous meteorological changes. This study introduces a distinctive perspective, focusing on AP, and seeks to evaluate the impact of different LULC conditions on the thermal comfort of urban residents. Moreover, we propose a conceptual model based on the relationship between AT/AP and LULC, aimed at providing invaluable insights for urban land use planning in the context of China's ongoing urbanization process, with the ultimate goal of enhancing the thermal comfort of urban residents and reducing city operating costs.

## Materials and methods

### Data collection

The meteorological data utilized in this study are obtained from the China Meteorological Science Data Sharing Service Center (http://data.cma.cn/) via the Daily Surface Climate Data Set of China. The dataset comprises a total of 834 meteorological stations across China, covering the period from 1996 to 2020. The data primarily includes variables such as daily mean air temperature (℃), atmospheric pressure (hPa), relative humidity (%), surface temperature (℃), wind speed (m/s), and precipitation (mm). All data have been subjected to quality control procedures by identifying and removing any outliers or erroneous records to ensure the accuracy and reliability. Any missing data are imputed through the use of interpolation techniques.

The land use and land cover data for China between 1996 and 2020 are obtained from the China Land Cover Dataset (CLCD) by Yang et al.^38^, using first-level classification raster data with a resolution of 30 m × 30 m.

The Digital Elevation Model (DEM) data for China with a spatial resolution of 500 m is obtained from the Resource and Environment Science and Data Center (RESDC) (http://www.resdc.cn/data.aspx?DATAID=123). This dataset originates from the Shuttle Radar Topography Mission (SRTM) data of the Endeavour space shuttle, which exhibits high accuracy and realistic representation.

The administrative division data is from the free and open data provided by the Resource and Environment Science and Data Center of the Institute of Geography and Resources Science, Chinese Academy of Sciences (https://www.resdc.cn/).

### Methods

#### K-nearest neighbor imputation

The K-Nearest Neighbors (KNN) algorithm is a simple and effective method for imputing missing data that has been widely used in various fields, including machine learning, pattern recognition, and data mining^39–41^. It works by identifying k observations with the shortest distances (usually based on Euclidean distance) from the missing values, and then replacing the missing values with weighted averages of the k nearest neighbors using distance-based inverse weighting. The procedure can be summarized in the following steps:

(1) Preprocess the data and establish a database.

(2) Calculate the Euclidean distances between the target data and other data points.

1 $$d(x,y) = \sqrt {(x_{1} - y_{1} )^{2} + (x_{2} - y_{2} )^{2} + \cdots + (x_{n} - y_{n} )^{2} } = \sqrt {\sum\limits_{i = 1}^{n} {(x_{i} - y_{i} )^{2} } }$$

(3) Sort the distances and select the k-nearest neighbors to the target data. It should be noted that the selection of k is a critical decision that balances model complexity and generalization. A smaller value of k leads to a more complex model that is prone to overfitting, while a larger value of k leads to a simpler model that is prone to underfitting. Generally, we chose a relatively small value of k based on the distribution of the samples, followed by cross-validation to determine the optimal value of k.

(4) Calculate the weights of each nearest neighbor (*w*_*i*_), which reflect the similarity between the target data and the selected neighbors.

2 $$w_{i} = \frac{{1/d_{i} }}{{\sum\limits_{i = 1}^{k} {1/d_{i} } }}$$

(5) Estimate the target data and perform imputation using the weighted average of the selected neighbors.

3 $$c_{x} = \sum\limits_{i = 1}^{k} {w_{i} c_{i} }$$

where* c*_*x*_ is the predicted value of the missing test data and *c*_*i*_ is the value of the nearest neighbor.

#### Apparent temperature

As apparent temperature (AP) is an empirical value that incorporates multiple meteorological parameters, there is no universally accepted method for its calculation. The method proposed by Steadman^16^ is currently the most widely accepted method for calculating AP. The formula is as follows:

4 $$AP = 0.33e + 0.70\; WS - 4.00$$

5 $$e = \frac{RH}{{100}} 6.105\quad \exp \left( {\frac{17.27 AT}{{237.7 + AT}}} \right)$$

where *AP* represents the apparent temperature, *e* represents the saturated vapor pressure of water, *WS* represents the wind speed, *RH* represents relative humidity, and *AT* represents the air temperature in degrees Celsius.

#### Time series analysis

Time series analysis is a widely used method for examining the periodic changes in variables^42,43^. In this study, we utilized the multiplicative model in time series seasonal analysis to analyze the AT and AP time series data across all meteorological stations. This model employs the moving average (MA) method to decompose time series into three components: trend, seasonality, and cyclical irregularity. The mathematical expression of the multiplicative model is given as:

6 $$Y_{t} = T_{t} \times S_{t} \times I_{t}$$

where *Y*_*t*_ represents the analyzed component at time* t*, *T*_*t*_ represents the trend component at time *t*, which reflects the long-term progression of the series (secular variation), *S*_*t*_ represents the seasonal component at time* t*, reflecting seasonality (seasonal variation), and *I*_*t*_ represents the irregular component (or "noise") at time *t*, which describes random, irregular influences.

Firstly, the moving average method is employed to identify the trend in the time series, followed by its elimination. Secondly, seasonal variations are determined by computing the mean of each period across all seasonal cycles. Lastly, the irregular component is obtained by filtering the trend and seasonal data from the original time series.

After obtaining the temperature trend data for each meteorological station using the multiplicative model, we performed a linear regression analysis on the data. In this analysis, temperature is treated as the dependent variable and time is treated as the independent variable. The aim of this analysis is to determine the rate of change in AT and AP for each station.

#### Inverse distance weight interpolation

Inverse Distance Weighted (IDW) interpolation method is a deterministic interpolation method that uses the data of known meteorological stations in the study area to predict the values of the unknown locations. The IDW method operates on the principle that points in close proximity have a greater influence on the interpolated value than those further away. It assumes that each sample point has a weight that decreases with increasing distance from the interpolated point. The closer a sample point is to the interpolated point, the greater its weight. When the sample point is beyond a certain distance, its weight can be ignored^44,45^. The principle of the IDW interpolation method can be expressed by the following formula:

7 $$z(x,y) = \sum\limits_{i = 1}^{N} {w_{i} T_{i} }$$

8 $$w_{i} = \frac{{d_{i}^{ - \alpha } }}{{\sum\limits_{i = 1}^{N} {d_{i}^{ - \alpha } } }}$$

where *z*(*x,y*)is the interpolated value at point (*x,y*)*, T*_*i*_ is the known value at the *i-*th sample meteorological station, *N* is the total number of sample meteorological stations, *w*_*i*_ is the weight of the *i*-th sample meteorological station,* d*_*i​*_ is the distance between the interpolated point and the *i*-th sample station, and *α* is a power parameter that determines how quickly the influence of a sample station decreases with distance, usually set to 2.

#### Land use extraction

It has been found that the heat stress effect is stronger in the core area of large cities with high population density^11^. Thus, within the QGIS Version 3.32.3 environment, the present study clips the shape file (Fig. S3) of the core urban area based on administrative divisions, building and road information, and actual urban conditions, and extracts land use data from the original dataset. This study primarily focuses on the land use patterns within the core areas of 30 developed cities, including some municipalities, provincial capitals, and sub-provincial cities. We define:

9 $$Ratio_{i} = \frac{{Area_{i} }}{{Area_{t} }}$$

where *Ratio*_*i*_ is the ratio of the area of *i*-type LULC to the total urban area, *Area*_*i*_ is the area of *i*-type LULC and *Area*_*t*_ is the total urban area.

#### Relationship between AP and LULC

To investigate the association between LULC and AP, it is reasonable to begin by examining the calculation methodology for AP. The formula for computing AP in the approach developed by Zhao^46^, which is based on the golden section method, can be expressed as follows:

10 $$AP = AT + A \times \left[ {\exp \left( {0.05 \times |T_{s} - AT| \times \left( {RH - RH_{s} } \right)} \right)} \right] + B \times |T_{s} - AT| \times WS$$

where *T*_*s*_ represents the optimal comfort temperature, *RH* represents relative humidity, *RH*_*S*_ represents Optimal comfort relative humidity, and *A* and *B* are coefficients.

This empirical equation can be transformed into the subsequent form with appropriate adjustments:

11 $$AP - AT - B \times |T_{s} - AT| \times WS = A \times \left[ {\exp \left( {0.05 \times |T_{s} - AT| \times \left( {RH - RH_{s} } \right)} \right)} \right]$$

12 $$\Delta T = A \times \exp (0.05 \times |T_{s} - AT| \times (RH - RH_{s} ))$$

13 $$\log (\Delta T) \propto |T_{s} - AT| \times (RH - RH_{s} )$$

where *∆T* represents *AP-AT*. As *T*_*s*_ and *RH*_*s*_ remain constant within a particular area, the formula can be further simplified to:

14 $$\log (\Delta T) \propto AT \times RH$$

According to the research conducted by Deng^27^, both urban expansion and lake shrinkage have been found to influence local temperature and humidity, ultimately affecting human thermal comfort. In some studies, to alleviate the urban heat island effect, it is commonly recommended to increase urban water areas and leverage the heat absorption process of water phase change to cool the temperature^47^. As previously mentioned, urban artificial surfaces, water bodies, and green spaces all have varying degrees of influence on meteorological factors, such as temperature and humidity, which subsequently affect human comfort perception^48–50^. Our analysis of land use patterns in 30 major cities revealed a significant correlation between the area of artificial surfaces and green spaces, with the two typically exhibiting an inverse relationship. By employing all-subsets regression method, we evaluated the impact of water area ratio (Wa_ratio), artificial surface area ratio (Ar_ratio), and green space area ratio (Gr_ratio) on ΔT, which indicates that the bivariate models comprising Wa_ratio and Ar_ratio, as well as those including Wa_ratio and Gr_ratio, both effectively evaluated ΔT with comparable effects (Fig. S4). Therefore, for the sake of model simplicity and accuracy, we select the bivariate model consisting of Wa_ratio and Ar_ratio to simulate ∆T in our study, as expressed by:

15 $$\Delta T \propto f(Wa\_ratio,\;Ar\_ratio)$$

Based on the spatial figures of ∆T ~ (Wa_ratio, Ar_ratio) and Eq. (14), we deduce that there exists a quadratic relationship between ∆T and the variables Wa_ratio and Ar_ratio. We then utilized the all subsets regression method to select the optimal subset of variables, and the model fitted by the least squares method is given below:

16 $$\Delta T = Intercept + B1 \times W_{a} \_ratio + B2 \times W_{a} \_ratio^{2} + B3 \times W_{a} \_ratio \times Ar\_ratio$$

where Intercept is the intercept, while *B1*, *B2* and *B3* are the respective regression coefficients.

## Results

### Spatial distribution of AT and AP

**Figure 1:**
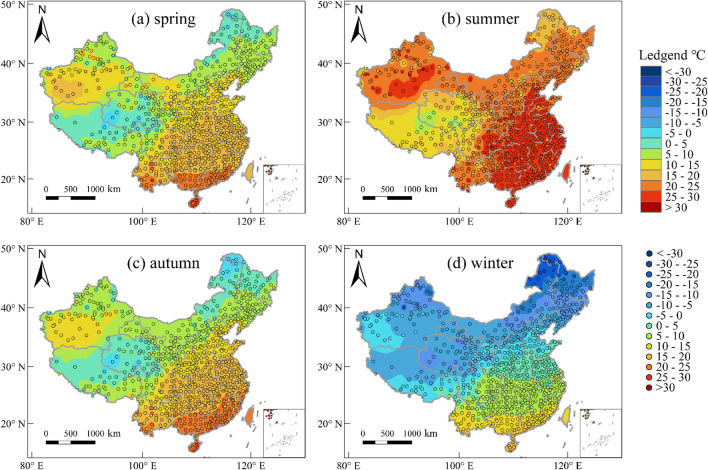
Distribution of AT in four seasons in China from 1996 to 2020. The maps were created using the Free and Open Source QGIS, Version 3.32.3 (https://www.qgis.org/en/site/).

This study investigates the spatial distribution of AT and AP across the four seasons, namely spring (March to May), summer (June to August), autumn (September to November), and winter (December to February). Figure 1 illustrates the spatial distribution of AT across different seasons in China over several years. The results indicate significant similarities between spring and autumn, except for the southern Taklamakan Desert region of Xinjiang, where the AT is marginally higher in spring. Conversely, during autumn, high-AT regions in southern China are more extensive than in spring. During the summer season, high-AT regions are distributed across North China, South China, East China, Central China, and certain areas of Hainan and Xinjiang, with the Turpan meteorological station in Xinjiang having the highest average AT of 32℃. Moreover, the Qinghai-Tibet Plateau, northern Inner Mongolia, and northern Heilongjiang remain cool in summer due to their high altitude and latitude, resulting in two distinct low-AT regions. The spatial distribution of AT in winter exhibits a clear north–south declining trend, with the lowest AT located at the boundary of Heilongjiang and northern Inner Mongolia, where the average AT can drop to as low as −20℃.

**Figure 2:**
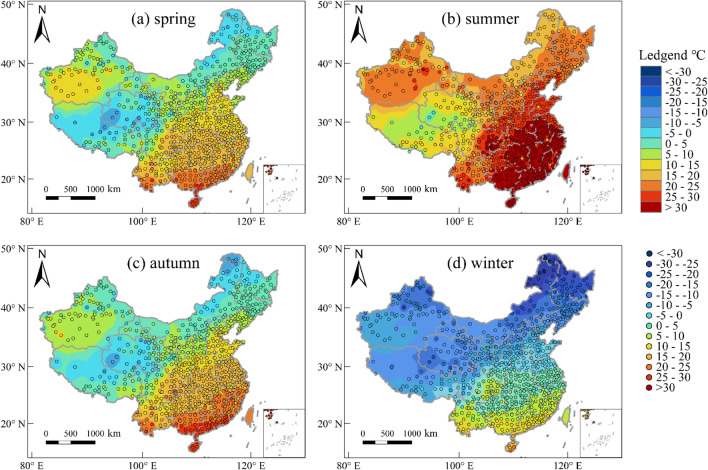
Distribution of AP in four seasons in China from 1996 to 2020. The maps were created using the Free and Open Source QGIS, Version 3.32.3 (https://www.qgis.org/en/site/).

Figure 2 illustrates the spatial distribution of the AP across different seasons in China over several years. During spring, autumn, and winter, the regions with high AP are mainly concentrated in the southern part of Hainan and Guangxi provinces, whereas the regions with low AP are primarily located in the Qinghai-Tibet Plateau, northern Inner Mongolia, and northern Heilongjiang Province. In contrast, during summer, the regions with high AP are widely distributed, primarily in the central, southern, and southeastern parts of China. It is notable that the spatial distribution of AP is substantially influenced by latitude and altitude. Specifically, as latitude increases, AP generally decreases from south to north. Similarly, as altitude increases, AP decreases from southeast to northwest.

Furthermore, the influence of the Qinling-Huaihe line on the AP is more pronounced than its effect on AT. This phenomenon may be attributed to the fact that AP is a composite climate index that takes into account various factors, including AT, wind speed, and humidity, whereas the Qinling-Huaihe line demarcates the boundary between the subtropical monsoon climate and the temperate monsoon climate in China^51^. As a result, the Qinling-Huaihe line serves as a fitting boundary for the cold and hot regions of the nationwide AP.

The spatial distribution of both AT and AP exhibits a high degree of overall similarity. However, the variance in the spatial distribution of AP surpasses that of AT, and the absolute values of extremums of low temperature and high temperature are also greater than those of AT. The spatial range of summer average AP between 30 and 35°C is considerably greater than that of AT, covering mainly the subtropical monsoon climate region of China. In a similar vein, the spatial range of low average AP (< 0°C) during winter is significantly larger than that of AT. The dividing line of winter average AT at 0°C lies to the north of the Qinling-Huaihe line. The winter average AT in Henan, southern Shandong, and central and southern Shaanxi exceeds 0°C. Nevertheless, the 0°C boundary of the winter average AP has shifted southward and distributed along the Qinling-Huaihe line. The spatial range of spring average AP > 15°C is smaller than that of AT. In southern Hebei, northern Henan, north-central Anhui, and southern Jiangsu, spring average AP is < 15°C, while AT in this area exceeds 15°C. The spatial distribution of both AT and AP in autumn is fundamentally similar, except that the range of average AP > 25°C is larger than that of AT, mainly concentrated in Hainan and the southern parts of Guangdong and Guangxi.

Overall, the use of AP reveals an increased spatial range of areas experiencing summer high temperatures (> 30°C) and winter low temperatures (< 0°C) compared to the corresponding AT data. Notably, some areas experience an increase in both high and low temperatures, which is mainly concentrated in the region between the Yangtze River and the Huai River, encompassing southern Henan, northern Wuhan, north-central Anhui, and north-central Jiangsu.

The distribution of temperature in China is also influenced by climatic zones. For instance, subtropical and tropical monsoon climate regions are the hottest areas in the country throughout the year, with average annual temperatures above 0°C. In contrast, plateau and alpine climates regions tend to be the coldest climate zones within the country. While temperate continental and temperate monsoon climate regions exhibit similar temperature ranges, the latter exhibits greater variability in temperature distribution. In addition, it must be noted that the spatial interpolation of AT and AP based on the IDW interpolation method may produce large errors in areas with relatively sparse meteorological stations and drastic changes in environmental factors such as altitude.

### Analysis of extreme cold and heat periods

In order to conduct an analysis of the periods of cold and heat periods in China, it is necessary to establish the temperature thresholds for cold and heat. Given the considerable differences in climatic conditions across the globe, there is no universally agreed-upon definition of heatwaves. Nonetheless, a range of temperature classification methods exist that cater to different research objectives. For instance, Brode, et al.^52^ defined strong thermal stress as an average temperature above 32℃, based on the human body's physiological response to heat. The World Meteorological Organization (WMO) recommends defining a heatwave as a period of at least three consecutive days with daily maximum temperatures exceeding 32 ℃ (90 °F). The Chinese commonly use the term "sauna weather" to describe high-temperature conditions and their effects on the body, which refers to weather with a relative humidity of exceeding 50% and AT exceeding 32℃. Furthermore, temperatures below the freezing point of water vapor (0℃) are generally considered low temperatures in meteorology, while meteorological departments typically calculate the number of cold days based on an average temperature below 0°C^53^. Recent studies have demonstrated that the occurrence of disease can escalate in the presence of extreme temperatures. While the threshold for extreme temperatures varies depending on the geographical location, an evaluation of the risk associated with extreme temperatures can be conducted through the computation of the spatial mean threshold, wherein the low temperature threshold is approximately 0°C and the high temperature threshold is approximately 32°C^5^. Based on this analysis, the high and low temperature thresholds are thus established as 32°C and 0°C, respectively.

#### The annual average days with extreme temperature

**Figure 3:**
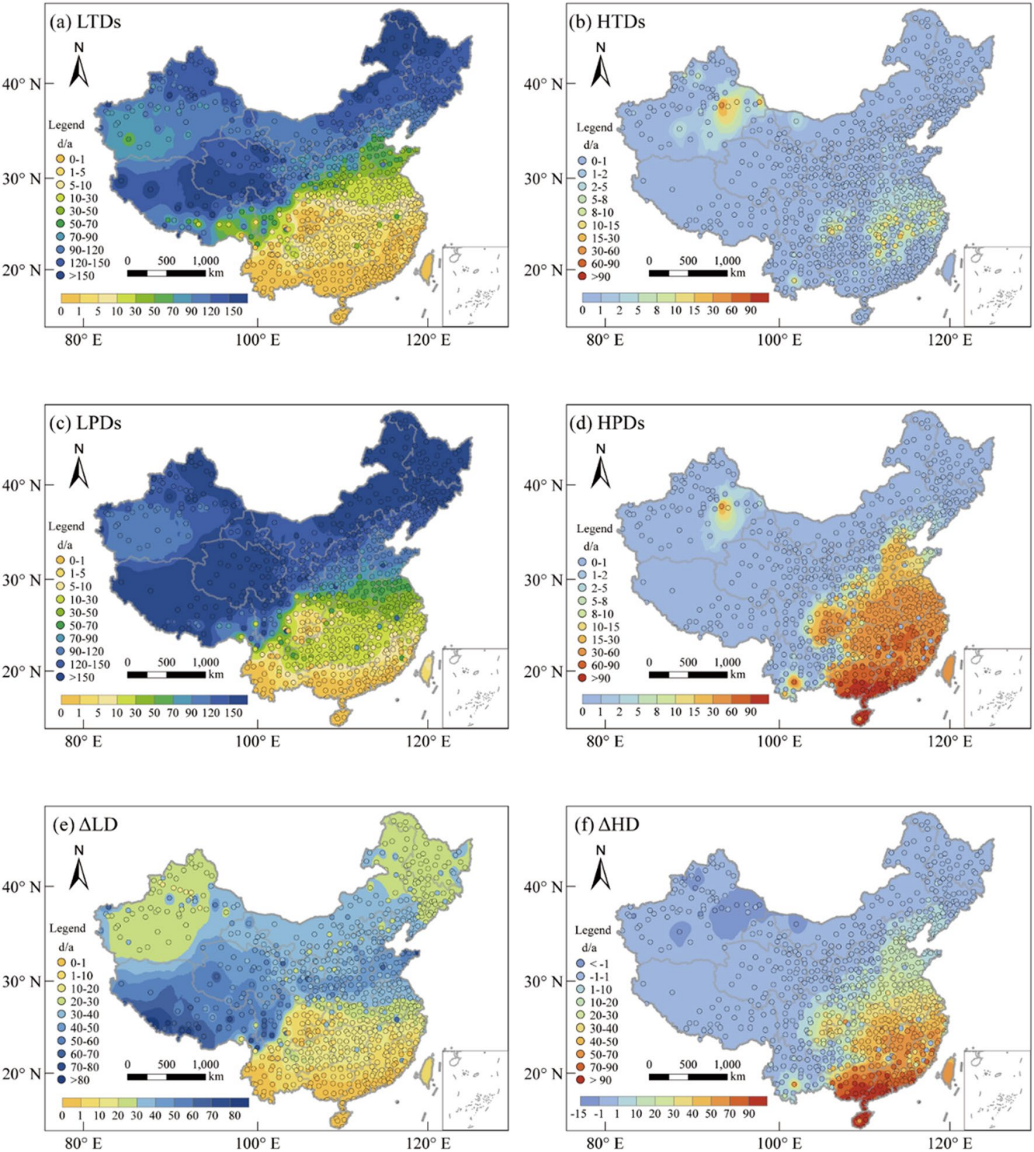
The spatial distribution of the annual average days with AT and AP, (**a**) number of low AT days (LTDs), (**b**) number of high AT days (HTDs), (**c**) number of low AP days (LPDs), (**d**) number of high AP days (HPDs), (**e**) difference of low temperature days (ΔLD: LPDs- LTDs), (**f**) difference of high temperature days (ΔHD: HPDs-HTDs). The maps were created using the Free and Open Source QGIS, Version 3.32.3 (https://www.qgis.org/en/site/).

This study presents a statistical analysis of the annual average days with extreme temperatures nationwide from 1996 to 2020, with the results depicted in Fig. 3. Figure 3a illustrates the spatial distribution of the annual average days with low AT (LTDs). The results indicate that regions with longer LTDs are predominantly concentrated in the Northeast, North China, Northwest, and Tibet, where LTDs exceeds 90 days per year. Notably, the Wudaoliang Meteorological Station in Qinghai Province records the longest LTDs in China, averaging 229 days per year over a 25-year period. Furthermore, LTDs ranges from 30 to 90 days per year in regions such as Shandong, Hebei, and southern Shanxi, whereas in other areas, LTDs is comparatively shorter. The southern region of the Qinling Mountains-Huaihe River line experiences less than 5 days per year with low temperatures. Figure 3b illustrates the spatial distribution of the annual average days with high AT (HTDs). The results indicate that regions with longer HTDs are mainly concentrated in the lower-middle reaches of Yangtze River Basin, and basically within a range below 30 days per year. In addition, some regions of Xinjiang also experience longer HTDs, with the Turpan meteorological station recording the longest HTDs in China, averaging 54.5 days per year. HTDs in other areas is relatively short.

Figure 3c illustrates the spatial distribution of the annual average days with low AP (LPDs). The overall spatial distribution pattern of LPDs is similar to that of LTDs, and the Wudaoliang meteorological station still records the longest LPDs in China, averaging 298 days per year. However, in contrast to LTDs, there has been a notable rise in LPDs in areas located north of the Yangtze River, where the majority of regions experience LPDs of over 50 days annually, and some even exceed 120 days per year. Meanwhile, the areas south of the Yangtze River still have a relatively short LPDs. Figure 3d illustrates the spatial distribution of the annual average days of high AP (HPDs). Unlike that of HTDs, the spatial distribution pattern of HPDs shows an obvious increasing trend from northwest to southeast. In most regions of Southern China, HPDs is more than 60 days per year, with some areas of southern Guangdong, southern Guangxi, and Hainan exceeding 90 days per year. The meteorological station in Xisha Islands, Hainan Province records the longest HPDs in China, with an average of 229 days per year. Compared to HTDs, HPDs has significantly increased in the areas south of the Yangtze River, with the degree of increase decreasing from south to north and from low altitude to high altitude.

#### Spatial distribution of ΔLD and ΔHD

According to the results, it can be concluded that there is a difference in the spatial distribution of the annual average days of extreme AT and extreme AP, with the latter generally having longer days than the former. These differences are expressed as ΔLD (LPDs- LTDs) and ΔHD (HPDs-HTDs), whose spatial distribution is shown in Fig. 3e, f, respectively. Figure 3e illustrates the spatial distribution pattern of ΔLD. The results indicate that the LPDs of the 834 meteorological stations surpass the LTDs. Moreover, the overall distribution pattern exhibits a distinct north–south gradient, with higher in the north and lower in the south. Notably, the junction of the Yellow River Basin and the JiangHuai region, as well as the Qinghai-Tibet Plateau, exhibit the highest values of ΔLD, indicating a significant disparity between LPDs and LTDs in these regions. Specifically, the Nyalam meteorological station in Tibet records the highest ΔLD value of 106 days per year. Figure 3f illustrates the spatial distribution of ΔHD. The results reveal that the East, Central and South China display higher values of ΔHD, with the ΔHD of Hainan and the southern regions of Guangdong and Guangxi typically exceeding 90 days per year. Notably, the Xisha meteorological station records the highest ΔHD value of 148 days per year. In contrast, the remaining regions exhibit lower ΔHD values, with the majority being below 5 days per year. In some areas of Xinjiang, the HTDs is higher than the HPDs.

#### Heat waves and cold waves

The aforementioned analysis reveals the distribution of the annual average days with extreme temperatures in China. However, it is worth noting that several consecutive days of extreme weather events, such as heat waves and cold waves, may potentially pose significant health and environmental risks^54–56^. According to the standards set forth by the China Meteorological Administration, when the daily maximum temperature exceeds 35 °C for a span of three consecutive days, it can be characterized as a “heat wave”. The definition of a cold wave is^57^: if the minimum temperature at a station drops by 8 °C within 24 h, or by 10 °C within 48 h, or by 12 °C within 72 h, and the daily minimum temperature falls below 4 °C, it is considered that a cold wave has occurred at that station.

**Figure 4:**
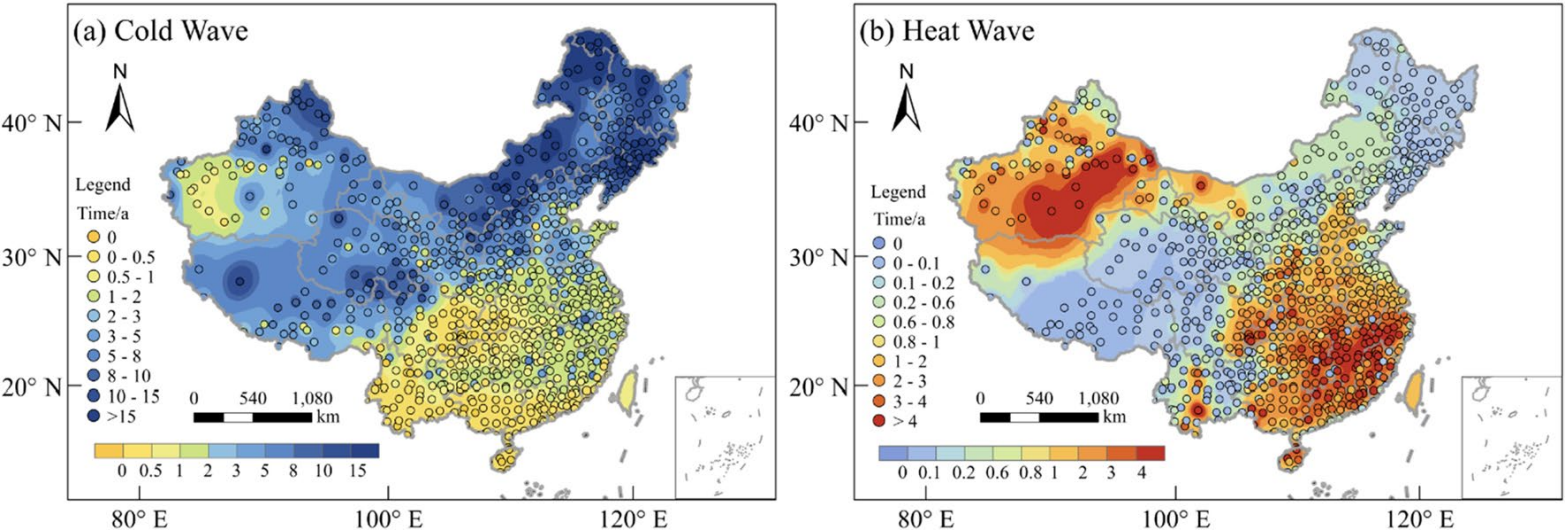
The spatial distribution of the annual average heat wave and cold wave frequency. The maps were created using the Free and Open Source QGIS, Version 3.32.3 (https://www.qgis.org/en/site/).

Figure 4a illustrates the spatial distribution of the annual average cold wave frequency in China from 1996 to 2020. The results indicate that cold waves occur more frequently in Northeast and Northwest China, which are also regions with low AP distribution in winter. According to the research findings, cold waves in China generally last for two to three days and rarely exceed seven days. From 1996 to 2020, approximately 3% of the 834 meteorological stations in China had not experienced a cold wave event, while around 30.6% of them had less than one cold wave event per year on average. About 29.4% of the stations experienced more than five cold wave events per year on average. Among these, approximately 13.2% of the stations had more than ten cold wave events per year on average. The location with the most frequent occurrence of cold waves is Tulihe Town in Inner Mongolia, with an average of 32 cold wave events per year, the longest of which lasted for six days and occurred in early November 2003.

Figure 4b illustrates the spatial distribution of the annual average heat wave frequency. The results reveal that heat waves occur more frequently in Southeastern China and Xinjiang, with Xinjiang exhibiting particularly frequent and prolonged instances of extreme heat waves. These areas also coincide with regions characterized by high AP in summer. According to the research findings, among the 834 meteorological stations in China, approximately 24.8% of these stations had not record any instances of heat wave events. Furthermore, around 33.6% of these stations had observed fewer than one heatwave event per year, while about 10% of the stations had reported an annual average of more than four heat wave occurrences. The most frequent incidence of heat waves is observed in Yuanjiang County, Yunnan, where the annual occurrence of heat waves reached as high as 13 times, with the longest lasting for 31 days, occurring in May 2019. But the Turpan region of Xinjiang had experienced the lengthiest duration of heat waves, with the most extended single event persisting for an astonishing 90 days during the summer of 2018.

Typically, heat waves last for approximately 3–4 days. However, in recent years, the frequency of heat waves has been steadily increasing, along with their duration. According to historical records, 31% of meteorological stations recorded their longest heatwave periods occurring after the year 2015.

### Long-term trends of AT and AP

**Figure 5:**
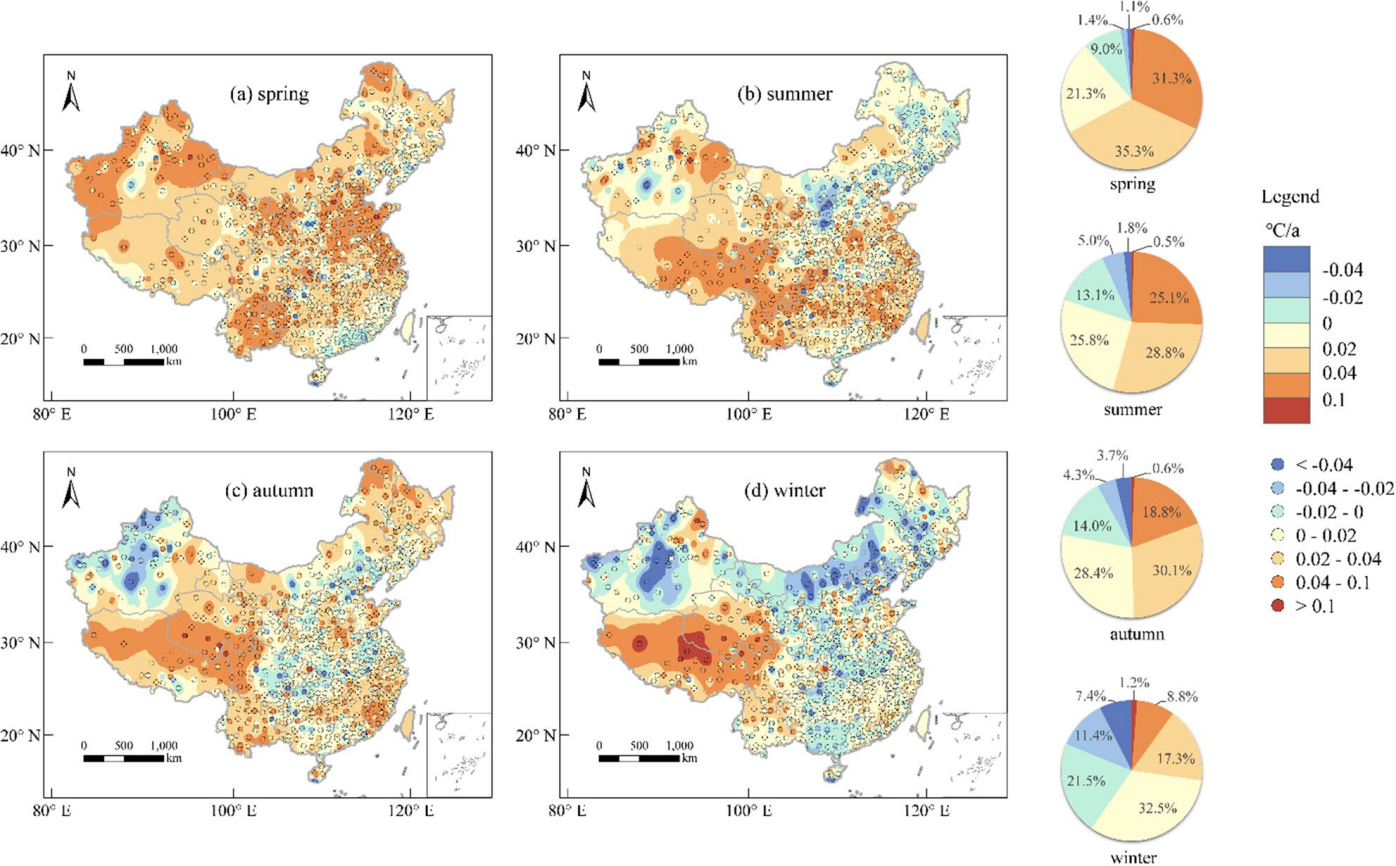
The spatial distribution of the annual average change rates of AT in four seasons. The pie chart represents the proportion of meteorological stations in the total across different AT change rates for each season. The maps were created using the Free and Open Source QGIS, Version 3.32.3 (https://www.qgis.org/en/site/).

**Figure 6:**
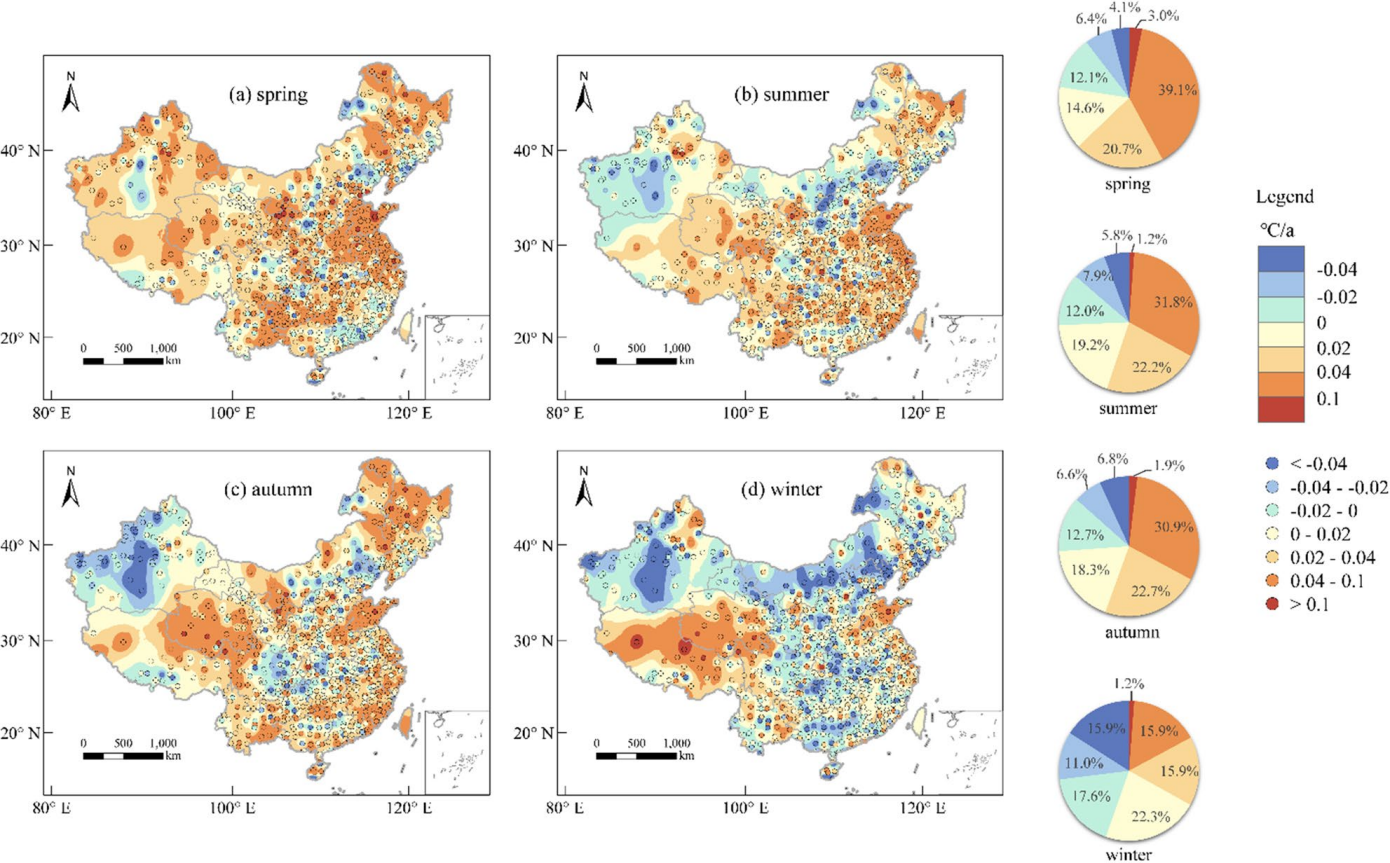
The spatial distribution of the annual average change rates of AP in four seasons. The pie chart represents the proportion of meteorological stations in the total across different AP change rates for each season. The maps were created using the Free and Open Source QGIS, Version 3.32.3 (https://www.qgis.org/en/site/).

In order to investigate the variation tendency in AT and AP across China from 1996 to 2020, this study conducts a time series analysis of temperature data over the 25-year period. We utilize the multiplicative model to extract the inter-annual trend of temperature changes by removing cyclic trends from temperature time series data. Subsequently, we employ a linear regression model to fit the relationship between temperature and time, in order to obtain the annual average change rates of AT and AP in different seasons across all meteorological stations. The results are illustrated in Figs. 5 and 6.

Figure 5 illustrates that there are significant variations in the spatial distribution of interannual temperature trends across different seasons, but overall, exhibiting a significant warming tendency. Among the four seasons, spring exhibits the most pronounced warming trend (Fig. 5a). Out of the 834 meteorological stations sampled, 738 stations (88.5% of the total) show an upward trend in AT, while 646 stations (77.5% of the total) display an ascending trend in AP. Summer (AT: 80.1%, AP: 74.3%) and autumn (AT: 77.9%, AP: 73.8%) also exhibit substantial warming trends, albeit less than spring. By contrast, winter manifests the weakest warming response, with only 59.7% of meteorological stations showing an upward trend in AT and 55.4% in AP. As a whole, the Qinghai-Tibet Plateau region represents the most prominent warming area in China, where all four seasons display an upward trend in temperature, with the majority of the region exhibiting an annual AT increase of over 0.02 ℃/a. Particularly, the winter season exhibits the most rapid warming rate, with certain areas exceeding 0.1 ℃/a. The regions with significant cooling trends vary in different seasons. Overall, the northern part of Shaanxi Province is one of the areas with the most significant cooling trend in China, especially in summer. And the winter season exhibits the broadest area of cooling. Distinct patterns of temperature trends can be observed in same areas across different seasons. In Xinjiang, for instance, there is a noticeable AT cooling trend in autumn and winter, while most areas display AT warming trends in spring and summer.

The spatial distribution of AP trend follows a pattern similar to that of AT trend. Nonetheless, the number of meteorological stations with a higher change rate of AP (> 0.04℃/a or < −0.04℃/a) is significantly greater than that of AT. There are 266 meteorological stations in spring, 213 in summer, 162 in autumn, and 83 in winter with an AT change rate > 0.04°C/a, while for AP, there are 351 meteorological stations in spring, 275 in summer, 274 in autumn, and 143 in winter with a change rate > 0.04°C/a. Moreover, there are 9 meteorological stations in spring, 15 in summer, 31 in autumn, and 62 in winter that show AT change rate < −0.04°C/a, while for AP, 34 meteorological stations in spring, 48 in summer, 57 in autumn, and 133 in winter exhibit an AP change rate < −0.04°C/a. As can be seen from Figs. 5 and 6, in the eastern China, the trend of change in AP is more significant than that of AT. In other words, the acceleration of warming and deceleration of cooling are both faster for AP than AT. Nevertheless, there are some regions where the trends in AT and AP exhibit opposite directions.

In conclusion, the overall variations of both AT and AP in China exhibit an upward trend. There are significant differences in spatial distribution in different seasons, and the trends of AP and AT changes also varies at the same location. Moreover, the range of high temperature change rates of AP is more extensive than that of AT.

### Relationship between temperature and LULC

**Figure 7:**
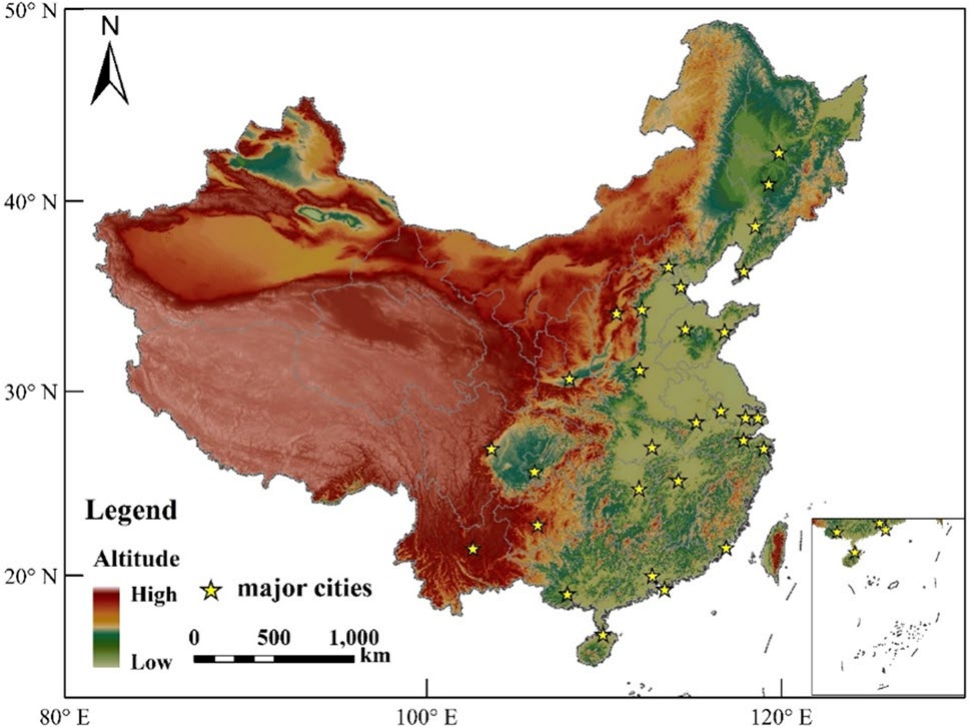
The spatial distribution of 30 major cities. The map was created using the Free and Open Source QGIS, Version 3.32.3 (https://www.qgis.org/en/site/).

**Figure 8:**
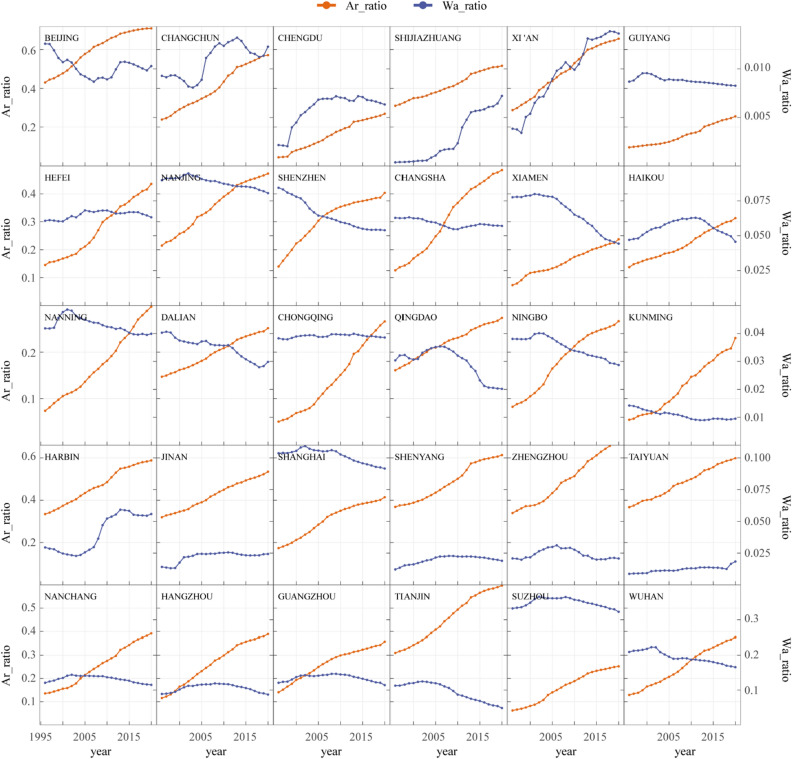
The ratio of water and artificial surface area in 30 cities from 1996 to 2020.

In recent years, there have been notable modifications in the national land use pattern, particularly with the acceleration of urban expansion. Urban water bodies have been replaced by buildings, roads, and other impervious paving, and phenomena such as land reclamation and lake reclamation are also common occurrences^22^. As AP is influenced by factors such as AT, RH, and WS, drastic LULC in large cities may alter local climatic conditions. For instance, a decrease in water area and green space may lead to an increase in urban temperature, while an increase in artificial surfaces may intensify the urban heat island effect, resulting in an increase in temperature^36^. In order to investigate the impact of urban land use and land cover (LULC) on temperature, this study has chosen to focus on 30 major cities in China, where the urban heat island effect tends to be more prominent due to high population density^11^ (Fig. 7). During the period from 1996 to 2020, urbanization in major cities in China has progressed rapidly, making it more likely to discover the relationship between land use and temperature using data from this period. We have extracted LULC data from the core areas of 30 cities spanning 1996 and 2020, with wetlands and water bodies being included as part of the water area. The ratio of water and artificial surface area in 30 cities from 1996 to 2020 is presented in Fig. 8 below (Fig. 8).

The intensified evaporation of water bodies and the significant heat absorption by artificial surfaces during summer high temperature conditions have a more pronounced effect on the urban heat index than under low temperature conditions, as evidenced by previous studies^58–60^. Consequently, this study utilizes a regression analysis approach to investigate the summer temperature of 30 major cities in China spanning the period between 1996 and 2020. The annual average change rates of summer AT and AP in 30 cities over the 25 years are shown in Table 1. Among the 30 major cities in China, 16 cities experience a significant warming trend (> 0.01°C/a) in summer AT, while 2 cities exhibit a significant cooling trend (< −0.01°C/a). The changes in the remaining cities are not significant, and there is no consistent trend between AP and AT changes.

**Table 1:** The annual average change rates of summer AT and AP in 30 cities from 1996 to 2020 (°C/a).

CITY	AT	AP	CITY	AT	AP
Beijing	0.013	0.054	Nanjing	0.033	0.020
Changchun	−0.003	0.036	Nanning	0.005	−0.027
Changsha	0.004	−0.010	Ningbo	0.008	0.017
Chengdu	0.029	0.031	Qingdao	0.028	0.080
Chongqing	0.079	0.057	Shanghai	0.030	0.048
Dalian	0.017	0.034	Shengyang	0.000	0.018
Guangzhou	0.003	0.011	Shenzhen	−0.001	0.048
Guiyang	−0.002	0.005	Shijiazhuang	−0.003	−0.021
Harbin	−0.006	0.029	Suzhou	0.043	0.074
Haikou	−0.011	−0.051	Taiyuan	0.007	−0.010
Hangzhou	0.044	0.036	Tianjin	0.032	0.024
Hefei	0.008	0.061	Wuhan	−0.024	−0.031
Jinan	0.015	0.030	Xiamen	0.045	0.051
Kunming	0.022	0.007	Xi 'an	0.029	0.048
Nanchang	0.061	0.048	Zhengzhou	0.076	0.062

**Figure 9:**
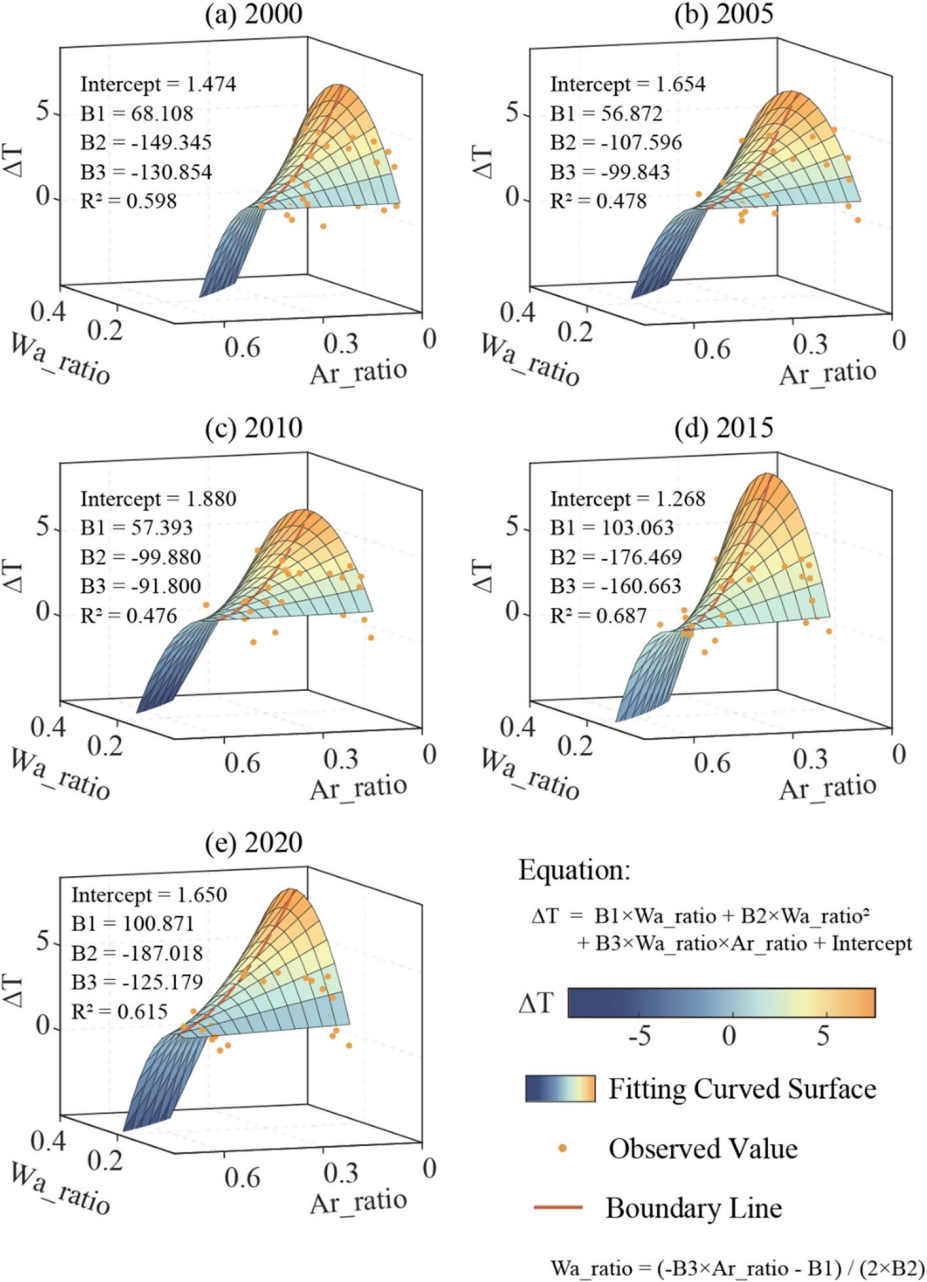
The relationship between ΔT and Wa_ratio and Ar_ratio in the core areas of cities. Where ΔT is the difference in AT and AP, Wa_ratio is the ratio of water area, and Ar_ratio is the ratio of artificial surface. Boundary Line is the dividing line which separate the ΔT changing trend into two segments, represent the least suitable LULC ratio relationship.

By incorporating the LULC data and the AP and AT data from the same period, the study has conducted a comprehensive analysis. The main aim of the analysis is to examine the relationship between the difference ΔT in AT and AP, and the ratios of water area (Wa_ratio) and artificial surface (Ar_ratio) in the core areas of the cities. The regression images of LULC and ΔT are shown in Fig. 9 (Full images are shown in Fig. S5(a)–(y)). Table S3 summarizes the regression results of each year, and their robustness is evaluated by Jackknife method.

The polynomial regression surface depicting the relationship of ΔT with respect to Wa_ratio and Ar_ratio exhibits a distorted downward parabolic shape, indicating the existence of a dividing line related to the ratio of water and artificial surface area. On the left side of the dividing line, ΔT shows an overall upward trend, whereas beyond the dividing line, it displays a downward trend. The partial derivatives of the two independent variables in Formula (16) are as follows:

Taking the partial derivative of Wa_ratio:

17 $$d(\Delta T)/d(W_{a} \_ratio) = B1 + 2 B2 W_{a} \_ratio + B3 Ar\_ratio$$

Taking the partial derivative of Ar_ratio:

18 $$d(\Delta T)/d(A_{r} \_ratio) = B3 \;W_{a} \_ratio$$

In order to obtain the dividing line, it is necessary to solve the equations:

19 $$d(\Delta T)/d(W_{a} \_ratio) = 0$$

20 $$d( \Delta T)/d(A_{r} \_ratio) = 0$$

Based on the graphical representation and the actual situation (Fig. 9), it can be inferred that the dividing line should be:

21 $$W_{a} \_ratio = ( - B3 Ar\_ratio - B1)/2B2$$

The average R^2^ value of the fitted results over multiple years is roughly 0.575, with the maximum R^2^ value of about 0.687 in 2015, and the minimum R^2^ value being approximately 0.466 in 2013. The *p*-values of the multi-year fitting analysis are generally less than 0.05, indicating that the bivariate quadratic relationship between ΔT and the Wa_ratio and Ar_ratio is statistically significant.

It should be noted that some variations remain inexplicable through the polynomial combination of Wa_ratio and Ar_ratio, which may include the effect of wind speed on AP. While simplifying Eq. (10), the impact of wind speed on AP has not been taken into account, but it has been considered during the regression analysis. Additionally, errors in land use data themselves, climate and altitude may also be the potential factors leading to fitting errors.

The aforementioned fitting analysis has effectively validated the hypothesis of Eq. (16), that there is a bivariate quadratic function relationship between ΔT and Wa_ratio and Ar_ratio. Furthermore, a boundary line exists on the downward parabolic surface, which separates the changing trend into two segments. Based on the fitting results obtained over multiple years, the relationship between Wa_ratio and Ar_ratio at the boundary line is approximately represented by the equation $W_{a} \_ratio = 0.263 - 0.437 \times Ar\_ratio$. This equation is derived by averaging the intercept and slope of the boundary line over several years, with standard deviations of 0.0269 and 0.0417, respectively. Consequently, it can be further speculated that the proportional relationship of $W_{a} \_ratio = 0.263( \pm 0.0269) - 0.437( \pm 0.0417) \times Ar\_ratio$ is the least suitable LULC ratio planning in the urban core area when accounting for the impact on the AP. We can call it the Least Suitable Curve (LSC).

## Discussions

In the current framework of weather assessment, AT is commonly employed as the primary metric for evaluating the local thermal comfort conditions^27,36,37^. Nevertheless, it is worth noting that in certain regions, the AP can deviate significantly from the AT due to the influence of meteorological factors such as wind speed and humidity^1,32,61^. According to the research results, we have identified some regions with significant differences between extreme AT and extreme AP. For instance, in the southeastern region of China, the number of extreme hot days quantified by AP is significantly higher than those measured using AT, consistent with previous research findings^33^. This discrepancy may be attributed to the frequent summer precipitation and the prevailing humid climatic conditions prevalent in these areas, thereby resulting in elevated AP values^34^. In previous studies, it has been observed that there are substantial disparities in the trends of extreme cold temperatures when considering the comprehensive temperature index compared to when neglecting it^13^. The current study identifies regions with significant disparities in extreme cold temperatures, revealing a pronounced difference between the extreme cold days assessed by AP and those evaluated using AT within the region spanning from the Qinghai-Tibet Plateau to the Shandong Peninsula. This dissimilarity can be attributed to the higher elevations and increased wind speeds prevalent in these areas, resulting in lower AP values during the winter season in this region. In these regions, the degree of hot (cold) discomfort from AP during summer (winter) surpasses the response measured solely by AT. It is difficult to accurately assess the thermal comfort relying solely on a single temperature indicator. Therefore, we recommend that when developing relevant regulations, both AT and AP should be considered comprehensively. Moreover, in Xinjiang Province, although the daily average temperatures during the summer are not exceptionally high, heat waves occur frequently. This phenomenon can be attributed to the substantial diurnal temperature variation in the area^62^. Daily average temperatures may not adequately capture the local thermal environment's complexity. Therefore, the use of daily maximum and minimum temperatures provides a more accurate reflection of the thermal comfort conditions in this region. The comprehensive consideration of multiple temperature indicators can assist in optimizing daily comfort and reducing the potential adverse consequences of extreme temperatures in people's lives.

The urban LULC change is recognized as a significant factor driving local climate variations, making it a crucial determinant affecting AP^11,25,26^. However, the mechanisms through which it exerts its influence are multifaceted and not limited to a singular pathway. Increasing urban water bodies is often regarded as an effective strategy for mitigating the urban heat island (UHI) effect in summer^27–29^, thereby achieving a reduction in urban AT. However, during this process, the phase transition of warm water into the atmosphere results in an elevation of atmospheric humidity^27,44,45^. The concurrent decrease in AT and increase in RH contribute to an ambiguous trend in AP. A similar uncertainty may exist in the relationship between artificial surface alterations and AP changes. The presence of these uncertainties serves as evidence of the diversity of pathways through which LULC change impacts AP. Based on the assumption of diverse impact pathways, a non-linear relationship between LULC and AP has been identified. The ridge line of the saddle-shaped surface represents the connecting line of local maxima, with the partial derivatives along the ridge line equating to zero (Fig. 9). Furthermore, the ridge line is conceptualized as the Least Suitable Curve (LSC), which is proposed in this study and provides a reliable perspective for formulating urban planning strategies. The LSC represents the relationship between Ar_ratio and Wa_ratio at which ΔT reaches its maximum value. Urban planning should establish rational LULC management objectives aimed at reducing AP, thereby striving to minimize ΔT in summer.

**Figure 10:**
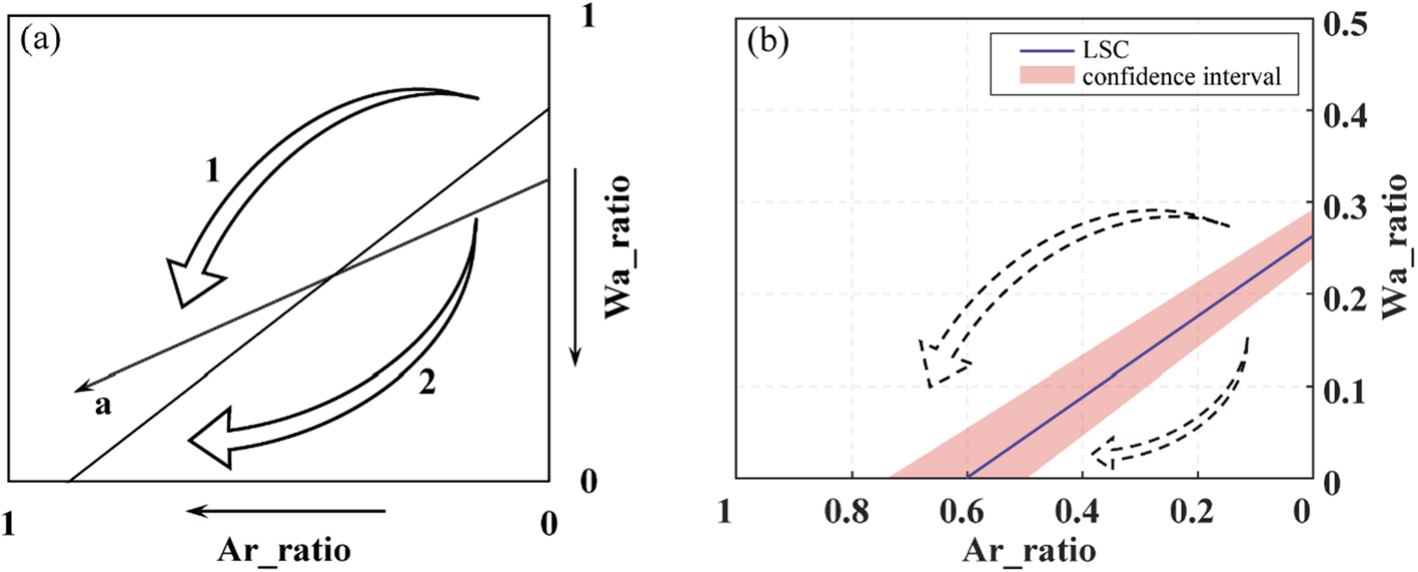
(**a**) The conceptual graph of how the Least Suitable Curve (LSC) guides urban planning. (**b**) The LSC predicted by this study.

When LSC is projected onto the Wa_ratio × Ar_ratio plane, the fitted non-linear patterns are further conceptualized (Fig. 10). Currently, urban development in China is primarily characterized by the expansion of artificial surfaces, the decline in lake areas, and the reduction of green spaces^21,22^. When undertaking future urban land use planning, it is essential to avoid the intersection of the planned urbanization paths, represented by changes in Wa_ratio and Ar_ratio, with the LSC (Path a in Fig. 10a), which would result in unnecessary wastage of resources. For instance, in cities where Wa_ratio is relatively low, if the primary approach to increase Ar_ratio involves encroaching upon non-water areas, this path may potentially intersect with the LSC. To optimize benefits, it is advisable to tailor the choice of a development path (e.g., Path 1 or 2) to align with the expected urban development. When Wa_ratio is relatively high, Path 1 becomes the more favorable option. Conversely, for cities with lower Wa_ratio, it is recommended to opt for Path 2. Notably, as shown in Fig. 10, in cities characterized by smaller Wa_ratio, it becomes paramount to maintain a harmonious Ar_ratio to ensure the thermal comfort of residents. In contrast to cities with a higher Wa_ratio, those with a lower Wa_ratio should target a more conservative optimal Ar_ratio. The projection of LSC discovered in this study onto the land use plane is illustrated in Fig. 10b.

Additionally, besides the LSC, attention should also be paid to the negative effects arising from an excessive proportion of a specific urban land use type. For instance, an increased proportion of industrial land may lead to an overall rise in urban temperature^36^. Therefore, blindly increasing or decreasing the area of a specific land use type should also be avoided during the planning process. The Least Suitable Curve proposed in this study can serve as a valuable reference. However, it is also crucial to comprehensively consider multiple factors when planning urban land use, in order to ensure a more rational approach that improves the thermal comfort of urban residents.

## Conclusions

Based on daily meteorological data from 834 sites and LULC data from the core areas of 30 developed cities across China from 1996 to 2020, this study investigates the spatiotemporal distribution and trends of AT and AP over a 25-year period, revealing the relationship between urban LULC changes and human thermal comfort. The main research findings are as follows:

1. The spatial distribution characteristics of AT and AP in China between 1996 and 2020 exhibit overall high similarity. However, the variance of AP spatial distribution is larger than that of AT.
2. A single temperature index is insufficient for assessing thermal comfort in regions with significant disparities between AT and AP. Regulatory systems should consider both AT and AP. In regions with significant diurnal temperature variations, it's also necessary to consider both the daily maximum and minimum temperatures.
3. Overall, both AT and AP show an increasing trend from 1996 to 2020 in China, with the most significant warming occurring in spring. The spatial heterogeneity of the trends is evident for both AT and AP, with more meteorological stations exhibiting a decreasing trend when using AP for trend analysis compared to AT. The spatial range of high AP change rates (> 0.04℃/a or < −0.04℃/a) is much wider than that of AT.
4. The warming trend is more pronounced in the core areas of larger cities. However, there is no consistent trend between AP and AT changes. The difference between the two variables, ΔT, is found to be affected by urban land use. There could exist a bivariate quadratic function relationship between ΔT and Wa_ratio and Ar_ratio. The study further reveals the potential existence of a least suitable curve (LSC), $W_{a} \_ratio = 0.263( \pm 0.0269) - 0.437( \pm 0.0417) \times Ar\_ratio$, which suggests that people tend to feel the most uncomfortable when the ratio of water area to artificial surface area is around this line. In the process of urban land use planning, based on a comprehensive analysis of climate and natural conditions, efforts should be made to avoid intersecting the designed paths with the Least Suitable Curve (LSC).

The findings reported here can offer valuable insights for urban land use planning, thereby improving the thermal comfort of urban inhabitants and reducing city operating costs.

### Supplementary Information

Supplementary Information.

## Data Availability

The datasets generated and/or analyzed during the current study are not publicly available due to the source data not being publicly available, but are available from the corresponding author on reasonable request.
