# Supplementary material for: Exploring the relationship between land use/land cover and apparent temperature in China (1996–2020): implications for urban planning
Source: Sci Rep. 2024 Feb 8;14:3214. doi: 10.1038/s41598-024-53858-8 (PMC10853208; doi:10.1038/s41598-024-53858-8)
Supplement: Supplementary file 1 — Supplementary Information. [file 41598_2024_53858_MOESM1_ESM.docx]

**Supplementary Materials**

**Title: Exploring the relationship between land use/land cover and apparent temperature in China (1996-2020): Implications for urban planning**

Han Ding^1,#^, Qiuru Ren^1,#^, Chengcheng Wang^1^, Haitao Chen^1^, Yuqiu Wang^1,*^

1 College of Environmental Science and Engineering, Nankai University, Tianjin 300350, China

* Correspondence:

Yuqiu Wang: [yqwang@nankai.edu.cn](mailto:yqwang@nankai.edu.cn)*.*

# These authors contributed equally to this work and should be considered co-first authors

**PART A: Performance evaluation of KNN method**

In our research, we obtained meteorological data from the China Meteorological Science Data Sharing Service Center (http://data.cma.cn/) through the Daily Surface Climate Data Set of China. This dataset contains specific codes with defined meanings. For example, code 32766 represents missing data, while code 32700 signifies trace amounts of precipitation, less than 0.1 mm, which typically has minimal impact on daily activities. For our calculations, we treated trace amounts as zero. Exceptional data points refer to values that are outliers, significantly deviating from the expected range for the respective meteorological parameter. For example, extremely high temperature readings in the thousands of degrees Celsius. When encountering such data, we initially assessed whether these anomalies resulted from decimal point misalignment or input errors. In cases of identified data errors, they were treated as missing data. Regarding the handling of missing data, which comprises less than 0.5% of the entire dataset, we employed interpolation using nearby meteorological data points for estimation. It is important to note that out of the 834 monitoring stations, 151 stations provided data only for the years 1996-2017, accounting for 18% of all stations. Data for the years 2018-2020 at these stations were interpolated based on the available data from 1996-2017.

To assess the effectiveness of the KNN interpolation method in data imputation, this study focused on Beijing as the research subject. Artificial missing values were introduced into the dataset covering the years 1996 to 2021. The missing data mechanism was set as Missing Completely At Random (MCAR), with a specified missing rate of 0.5%. The simFrame package in R was utilized to simulate the data missing process. To determine the most suitable value of k, the Normalized Root Mean Squared Error (NRMSE), coefficient of determination (R^2^), and Spearman rank correlation coefficient were employed to measure the goodness of fit between the interpolated results and the actual observed values. After conducting cross-validation, a final choice of k = 10 was made, and the fitting results are presented as follows:

Table S1. K-Nearest Neighbors Algorithm Imputation Results

| missing rate | parameter | Spearman | R^2^ | NRMSE |
| --- | --- | --- | --- | --- |
| 0.5% | TEM | 0.994 | 0.994 | 0.050 |
|  | RHU | 0.938 | 0.900 | 0.155 |
|  | WIN | 0.866 | 0.780 | 0.166 |
|  | GST | 0.987 | 0.985 | 0.067 |


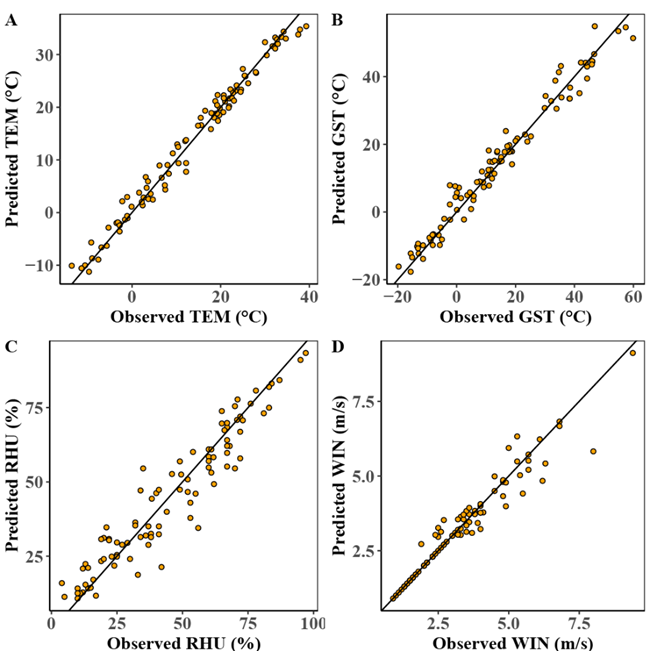


Fig.S1 Fit Plot of Predicted vs. Observed Values Using K-Nearest Neighbors Algorithm for 0.5% Missing Rate, where TEM is air temperature, GST is surface temperature, RHU is relative humidity, and WIN is wind speed.

Based on the results and analysis provided, it is evident that the KNN method is effective for imputing missing data, particularly for temperature, with satisfactory performance for humidity and wind speed. As a result, we have chosen to utilize the KNN interpolation method.

**PART B: Assessment of the Suitability of Additive and Multiplicative Models in Time Series Analysis**

In order to determine the most appropriate model – whether additive or multiplicative – for our study, we performed an analysis utilizing Beijing's summer temperature data for the years spanning 1996 to 2020 as a case study. We employed both the additive and multiplicative models to decompose the time series into its underlying trend and seasonal components. As observed in the decomposition results (Fig.S2), it is discernible that the variation in the error curve resulting from the multiplicative decomposition was considerably smaller when compared to that from the additive decomposition.


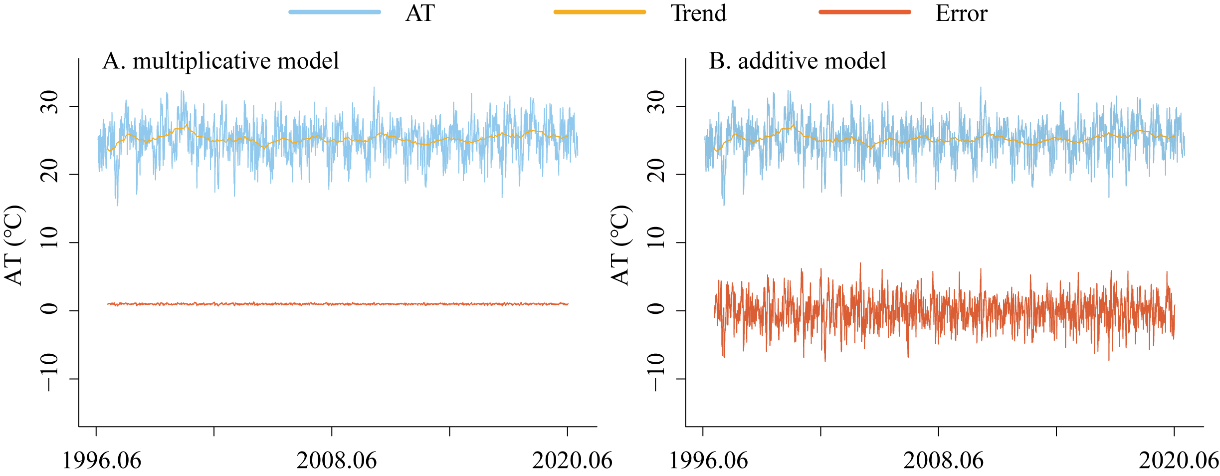


Fig.S2 Sequence comparison after decomposition of seasonal factor multiplication(a)and addition (b)

Further support for this observation was gleaned from the analysis of the error sequences. For the multiplicative decomposition, the maximum error value was 1.3, the minimum was 0.69, and the variance was 0.0076. In contrast, the additive decomposition yielded a maximum error value of 7.0, a minimum of -7.5, and a variance of 4.7. This comprehensive analysis conclusively favored the multiplicative decomposition approach^[1, 2]^.

Moreover, it is pertinent to note that the multiplicative model stands as a widely recognized and extensively utilized methodology in the realm of time series decomposition, particularly in the domain of meteorological data analysis^[3, 4]^.

**PART C: Land use extraction**

It has been found that the heat stress effect is stronger in the core area of large cities with high population density^[5]^. Thus, within the ArcGIS environment, the present study clips the shape file (Fig.S3) of the core urban area based on administrative divisions, building and road information, and actual urban conditions, and extracts land use data from the original dataset.

Fig.S3 Core areas of 30 major cities

**PART D:**


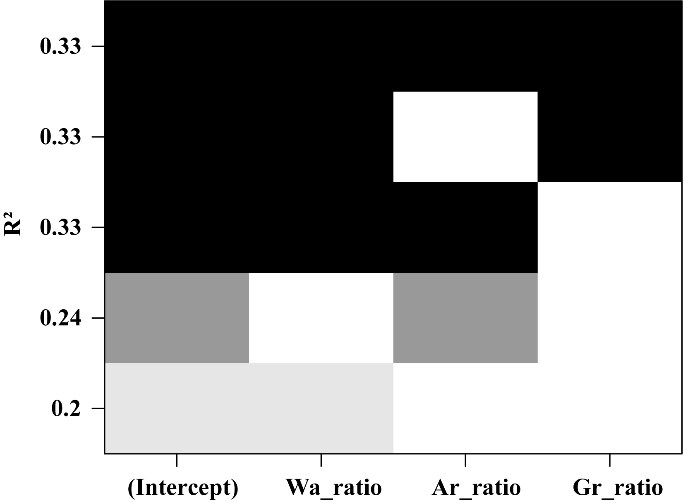


Fig.S4 Based on R^2^, the correlation between three types of land use and ∆T

**PART E: The regression fitting results of ΔT and Wa_ratio and Ar_ratio**

To assess the performance of the proposed model in this study, we employed the K-fold cross-validation method^[6, 7]^. K-fold cross-validation is a technique in model evaluation designed to efficiently utilize limited datasets. It divides the original dataset into K equally sized subsets, sequentially using each subset as a validation set while the remaining K-1 subsets serve as the training set. This process repeats K times, with each subset serving as the validation set exactly once. Ultimately, the evaluation of model performance is based on the average or summary of these K validation results. This method provides a more comprehensive assessment of model performance, mitigating evaluation bias caused by specific data partitions. In this study, we utilized the data from the year 2000 as an example for model (16) performance evaluation, with K set to 5^[8]^. The results are as follows:

$\text{∆T }\text{= }\text{Intercept + B1 }\text{}\text{ }\text{W}_{\text{a}}\_\text{ratio }\text{+ }\text{B2 }\text{}{\text{ W}_{\text{a}}\_\text{ratio}}^{\text{2}}+\text{B3 }\text{}\text{ }\text{W}_{\text{a}}\_\text{ratio }\text{}\text{ }\text{Ar\_ratio}$ (16)

Table S2 Fitting results of K-fold cross-validation method

|  | Mean | Standard Error | p value |
| --- | --- | --- | --- |
| Intercept | 1.474 | 0.012 | 0.00008 |
| B1 | 68.108 | 0.547 | 0.00001 |
| B2 | -149.345 | 2.284 | 0.00003 |
| B3 | -130.854 | 1.274 | 0.003 |

Upon analysis of the results, it is observed that the standard errors of the fitted coefficients in the model are relatively small. Furthermore, the p-values associated with these coefficients are significantly smaller than the conventional significance level, indicating that the parameter estimates possess a high degree of robustness. The same methodology was applied to fit the data for other years, and the results are as follows:

Table S3 Regression Results and Boundary Lines of Binary Quadratic Function

| year | Intercept | B1 | B2 | B3 | R^2^ | p-value | Boundary Line |
| --- | --- | --- | --- | --- | --- | --- | --- |
| 1996 | 1.569 | 68.651 | -159.801 | -164.730 | 0.579 | 4.27×10^-5^ | Wa_ratio=0.215-0.515×Ar_ratio |
| 1997 | 1.232 | 69.646 | -161.608 | -143.256 | 0.594 | 2.69×10^-5^ | Wa_ratio=0.215-0.443×Ar_ratio |
| 1998 | 1.826 | 64.567 | -148.265 | -131.022 | 0.527 | 1.84×10^-4^ | Wa_ratio=0.218-0.442×Ar_ratio |
| 1999 | 1.430 | 71.969 | -165.890 | -146.732 | 0.596 | 2.53×10^-5^ | Wa_ratio=0.217-0.442×Ar_ratio |
| 2000 | 1.474 | 68.108 | -149.345 | -130.854 | 0.598 | 2.38×10^-5^ | Wa_ratio=0.228-0.438×Ar_ratio |
| 2001 | 1.225 | 75.489 | -162.388 | -138.913 | 0.662 | 2.60×10^-6^ | Wa_ratio=0.232-0.428×Ar_ratio |
| 2002 | 1.272 | 79.665 | -163.280 | -155.873 | 0.607 | 1.77×10^-5^ | Wa_ratio=0.244-0.477×Ar_ratio |
| 2003 | 1.320 | 74.688 | -149.029 | -139.116 | 0.594 | 2.73×10^-5^ | Wa_ratio=0.251-0.467×Ar_ratio |
| 2004 | 1.227 | 70.091 | -135.097 | -127.301 | 0.585 | 3.60×10^-5^ | Wa_ratio=0.259-0.471×Ar_ratio |
| 2005 | 1.654 | 56.872 | -107.596 | -99.843 | 0.478 | 6.45×10^-4^ | Wa_ratio=0.264-0.464×Ar_ratio |
| 2006 | 1.551 | 61.612 | -115.291 | -98.083 | 0.566 | 6.35×10^-5^ | Wa_ratio=0.267-0.425×Ar_ratio |
| 2007 | 1.587 | 64.274 | -113.217 | -110.116 | 0.510 | 2.91×10^-4^ | Wa_ratio=0.284-0.486×Ar_ratio |
| 2008 | 1.606 | 57.813 | -104.785 | -88.636 | 0.520 | 2.21×10^-4^ | Wa_ratio=0.276-0.423×Ar_ratio |
| 2009 | 1.265 | 78.018 | -135.998 | -129.349 | 0.600 | 2.26×10^-5^ | Wa_ratio=0.287-0.476×Ar_ratio |
| 2010 | 1.880 | 57.393 | -99.880 | -91.800 | 0.476 | 6.66×10^-4^ | Wa_ratio=0.287-0.460×Ar_ratio |
| 2011 | 1.558 | 60.177 | -107.771 | -81.352 | 0.531 | 1.67×10^-4^ | Wa_ratio=0.279-0.377×Ar_ratio |
| 2012 | 1.640 | 71.380 | -121.273 | -110.023 | 0.569 | 5.68×10^-5^ | Wa_ratio=0.294-0.454×Ar_ratio |
| 2013 | 1.822 | 69.076 | -116.978 | -108.357 | 0.466 | 8.50×10^-4^ | Wa_ratio=0.295-0.463×Ar_ratio |
| 2014 | 1.320 | 101.860 | -176.560 | -154.852 | 0.646 | 4.60×10^-6^ | Wa_ratio=0.288-0.439×Ar_ratio |
| 2015 | 1.268 | 103.063 | -176.469 | -160.663 | 0.687 | 1×10^-6^ | Wa_ratio=0.292-0.455×Ar_ratio |
| 2016 | 1.838 | 92.798 | -163.515 | -133.020 | 0.604 | 1.98×10^-5^ | Wa_ratio=0.284-0.407×Ar_ratio |
| 2017 | 1.608 | 104.659 | -184.385 | -148.874 | 0.631 | 7.90×10^-6^ | Wa_ratio=0.284-0.404×Ar_ratio |
| 2018 | 1.939 | 80.152 | -148.771 | -103.973 | 0.499 | 5.35×10^-4^ | Wa_ratio=0.269-0.349×Ar_ratio |
| 2019 | 1.460 | 113.318 | -202.967 | -160.906 | 0.624 | 1.64×10^-5^ | Wa_ratio=0.279-0.396×Ar_ratio |
| 2020 | 1.637 | 102.720 | -193.205 | -127.041 | 0.602 | 3.25×10^-5^ | Wa_ratio=0.266-0.329×Ar_ratio |


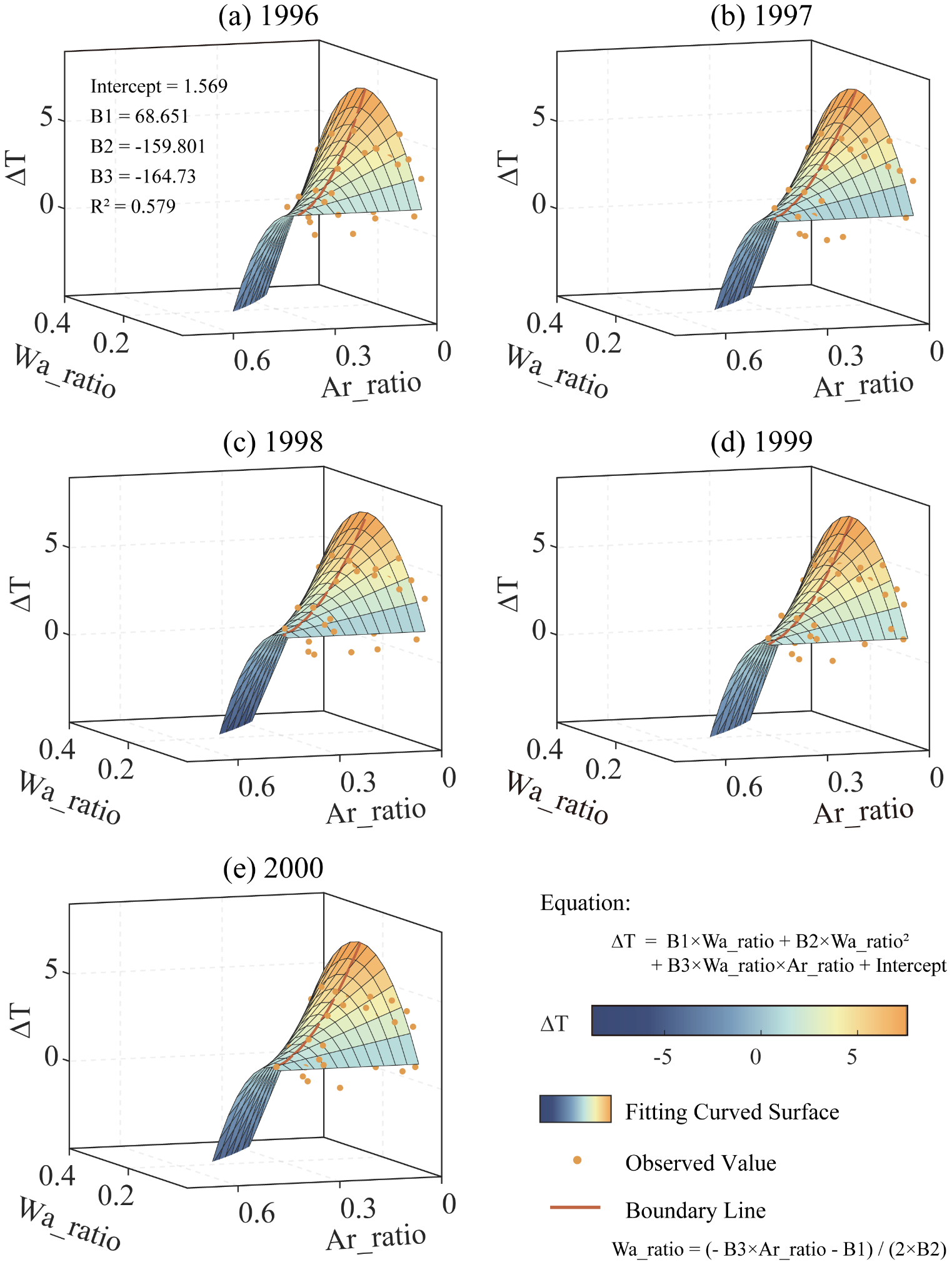

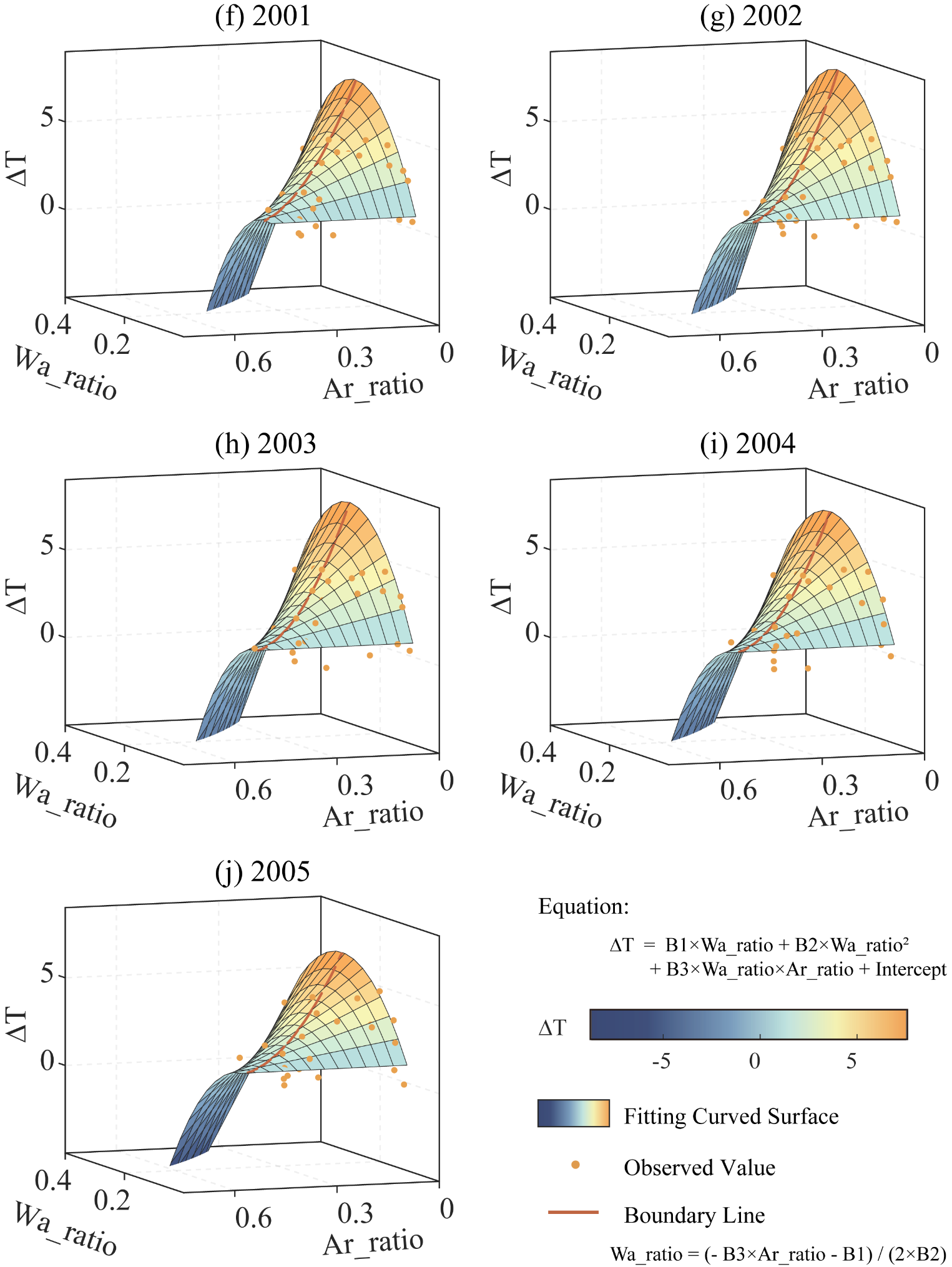

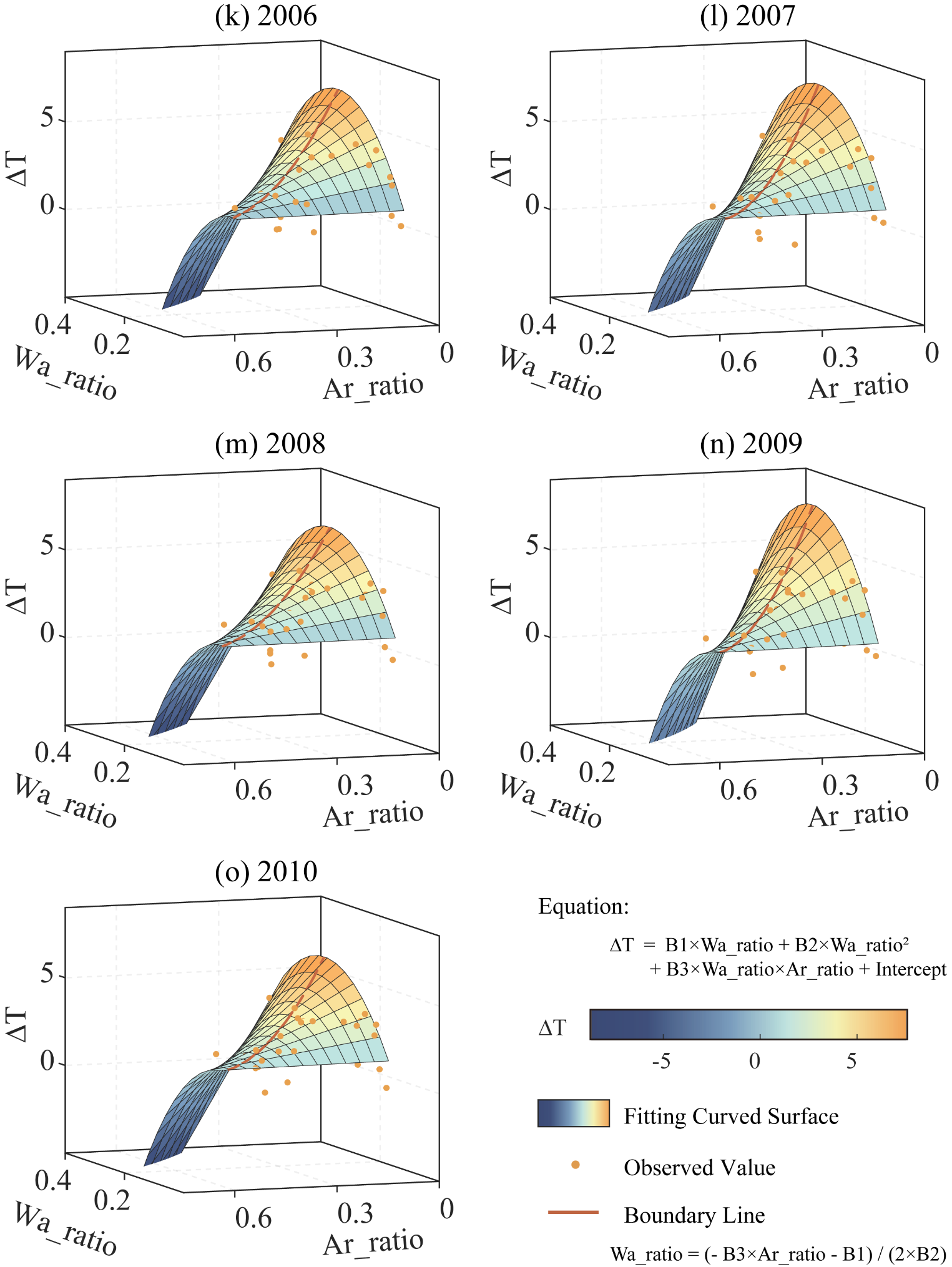

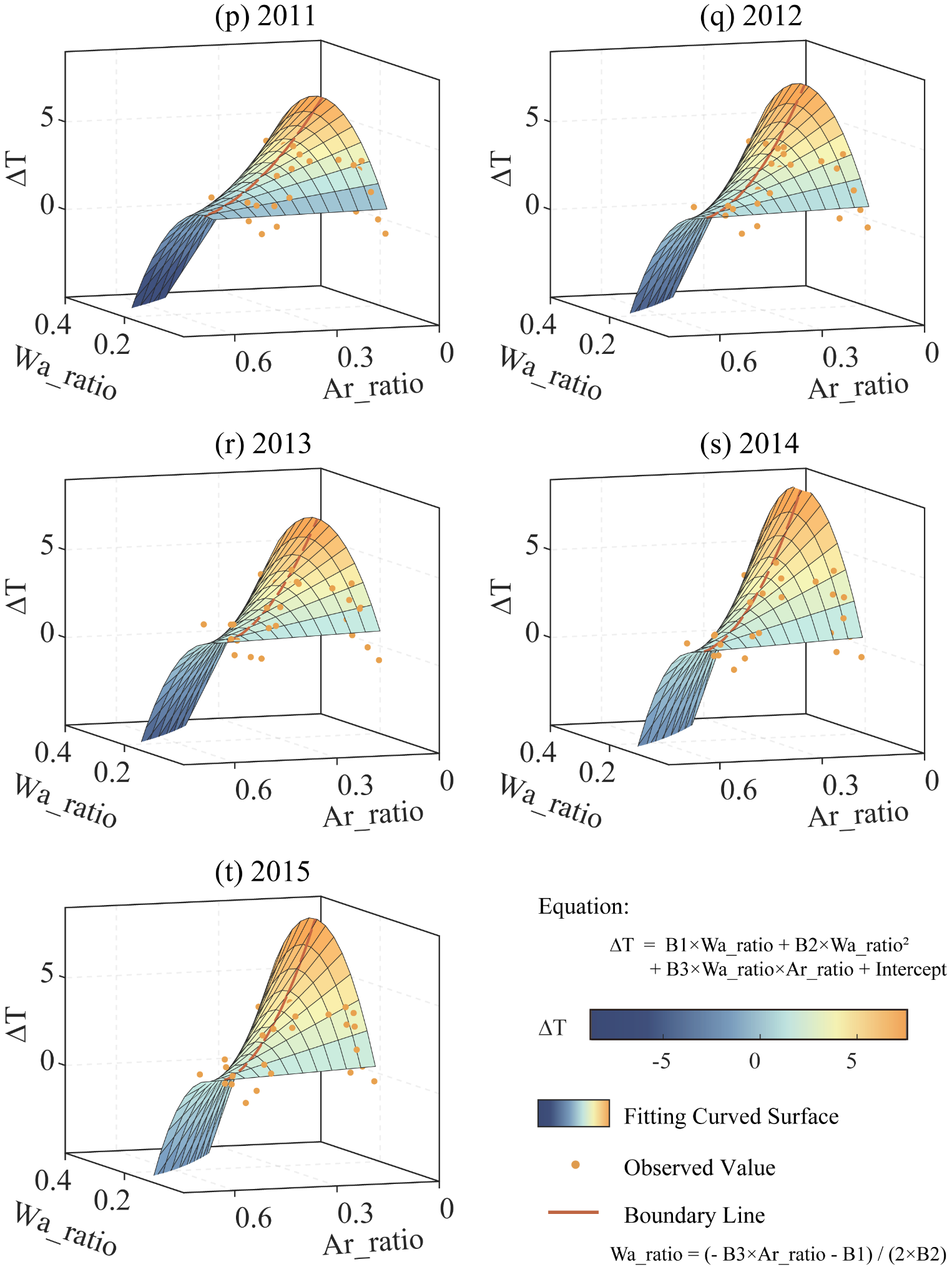

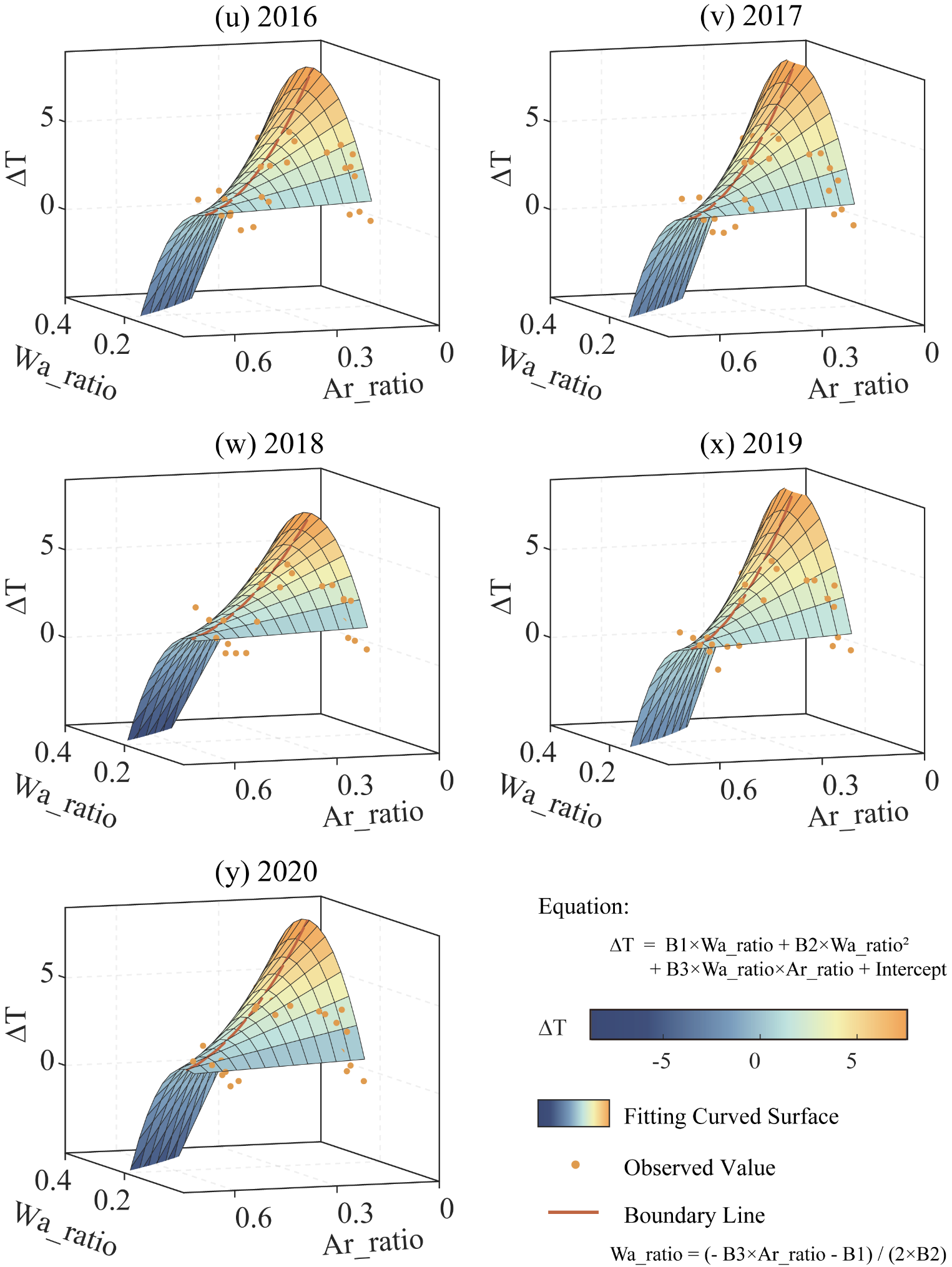


Fig.S5 The relationship between ΔT and Wa_ratio and Ar_ratio in the core areas of cities from 1996-2020.

**PART F: Abbreviations / Acronyms and Full Names Reference Table**

Table S3 Abbreviations / Acronyms and Full Names Reference Table

| Abbreviation / Acronym | Full name |
| --- | --- |
| AT | air temperature |
| AP | apparent temperature |
| ΔT | AP-AT |
| LULC | land use/land cover |
| Wa_ratio | ratio of water area to urban area |
| Ar_ratio | ratio of artificial surface to urban area |
| Gr_ratio | ratio of green space to urban area |
| RH | relative humidity |
| LTDs | the annual average days with low air temperature |
| HTDs | the annual average days with high air temperature |
| LPDs | the annual average days with low apparent temperature |
| HPDs | the annual average days of high apparent temperature |
| ΔLD | LPDs- LTDs |
| ΔHD | HPDs- HTDs |
| LSC | the Least Suitable Curve |

**Reference**

[1] Wen K, Cui P, Yang H*, et al.* The relationship between ENSO and the the precipitation extremes along the middle reaches of Yangtze River during the monsoon season. *Mountain Research (in Chinese)*, 2011, 29:299-305.

[2] Tan R, Wang C, Chen R. Time series analysis and forecast of rainfall in Nanning City based on the rainfall data during the last 13 years. *Journal of Anhui Agricultural Sciences (in Chinese)*, 2022, 50:191-193+197.

[3] Palagin ED, Gridneva MA, Bykova PG*, et al.* Urban land: study of surface run-off composition and its dynamics. International Conference on Construction, Architecture and Technosphere Safety (ICCATS). Chelyabinsk, RUSSIA, 2017.

[4] Olszanka A, Dittrichová J. Direction of changes on the milk markets in Poland, the Czech Republic and Slovakia in comparison with the biggest milk producers in EU (2005-2016). 16th International Scientific Conference on Hradec Economic Days. Hradec Kralove, CZECH REPUBLIC, 2018. pp. 92-101.

[5] Luo M, Lau NC. Increasing human-perceived heat stress risks exacerbated by urbanization in China: A comparative study based on multiple metrics. *Earth's Future*, 2021, 9:e2020EF001848.

[6] Wong TT, Yeh PY. Reliable accuracy estimates from k-fold cross validation. *Ieee Transactions on Knowledge and Data Engineering*, 2020, 32:1586-1594.

[7] Vu HL, Ng KTW, Richer A*, et al.* Analysis of input set characteristics and variances on k-fold cross validation for a Recurrent Neural Network model on waste disposal rate estimation. *Journal of Environmental Management*, 2022, 311.

[8] Yu ZX, Huang F, Zhao XH*, et al.* Predicting drug-disease associations through layer attention graph convolutional network. *Briefings in Bioinformatics*, 2021, 22.
